# Impact of Increasing Levels of Oxygen Consumption on the Evolution of Color, Phenolic, and Volatile Compounds of Nebbiolo Wines

**DOI:** 10.3389/fchem.2018.00137

**Published:** 2018-04-27

**Authors:** Maurizio Petrozziello, Fabrizio Torchio, Federico Piano, Simone Giacosa, Maurizio Ugliano, Antonella Bosso, Luca Rolle

**Affiliations:** ^1^Consiglio per la ricerca in agricoltura e l'analisi dell'economia agraria, Centro di Ricerca Viticoltura ed Enologia, Asti, Italy; ^2^Istituto di Enologia e Ingegneria Agro-Alimentare, Università Cattolica del Sacro Cuore, Piacenza, Italy; ^3^Dipartimento di Scienze Agrarie, Forestali e Alimentari, Università degli Studi di Torino, Turin, Italy; ^4^Dipartimento di Biotecnologie, Università di Verona, Verona, Italy

**Keywords:** oxygen, Nebbiolo, wine, CIEL^*^a^*^b^*^ color, aldehydes, wine aging, tannins

## Abstract

Since the end of the last century, many works have been carried out to verify the effect of controlled oxygen intake on the chemical and organoleptic characteristics of red wines. In spite of the large number of studies on this subject, oxygen remains a cutting-edge research topic in oenology. Oxygen consumption leads to complex and not univocal changes in wine composition, sometimes positive such as color stabilization, softening of mouthfeel, increase of aroma complexity. However, the variability of these effects, which depend both on the oxygenation conditions and the composition of the wine, require more efforts in this research field to effectively manage wine oxygen exposure. The present study is focused on the evolution of the chemical composition of four different Nebbiolo wines, each of them added with 4 different doses of oxygen (7, 14, 21, and 28 mg/L total intake) during the first month of storage. In this perspective, the evolution over time of wine color and polyphenols was studied. Acetaldehyde, glyceraldehyde and glyoxylic acid were quantified by HPLC. These compounds can play a role in wine aging creating condensed colored and stable products involving anthocyanins with or without tannins. Moreover, some volatile aldehydes correlated with oxidized olfactory notes, including methional and (E)-2-alkenals, have been quantified by GC-MS. Overall, during storage a decrease of color intensity, total and free anthocyanins and an increase in polymeric pigments (in particular the contribution to the red color of pigments not-bleachable by SO_2_ or dTAT%) and some minor aldehydes was observed. Nevertheless, the differences in color parameters between the samples with different doses of oxygen were modest. These evidences were in contrast with an evident and detectable increase of free acetaldehyde content at increasing doses of oxygen measured after 60 days of storage. The effect of oxygen on color and production of SO_2_ non-bleachable pigments during aging varies with wine composition, with Nebbiolo wines appearing not very reactive in this respect, probably due to their low content in anthocyanins and high content in tannins.

## Introduction

The role of oxygen in winemaking has been studied for more than a century, since the early works of Berthelot (1863) and Pasteur (1866). However, its contribution to wine quality remains in part controversial. Oxidative spoilage resulting from excessive exposure of the wine to oxygen represents nowadays a major issue for the wine industry. In recent years there has been increasing concern about oxidation problems, not only in white wines (Bessis, 2007) but also in red wines (Pons et al., 2013). At the same time, in agreement with the work of Pasteur, many recent studies have confirmed that a moderate exposure to oxygen during either winemaking or bottle storage can have several benefits to wine technological and sensory quality, including increased color stability, improved mouthfeel and aroma complexity as well as elimination of reductive off-odors (Wirth et al., 2010, 2012; Ugliano et al., 2012; Gambuti et al., 2013).

Wine exposure to oxygen induces the rapid transformation of the most oxidizing compounds, in particular polyphenols. Wildenradt and Singleton (1974) demonstrated that the oxidation of vicinal di– and tri–hydroxyphenols leads to the formation of quinones and hydrogen peroxide. These compounds can oxidize other substances, including ethanol as the most abundant and organic acids. This process leads formation of different aldehyde such as acetaldehyde, glyceraldehyde, and glyoxylic acid, which derive from the oxidation of ethyl alcohol, glycerol, and tartaric acid respectively (Grant-Preece et al., 2017). Acetaldehyde is the most abundant aldehyde compound in wine, at high levels may be associated with unpleasant odors of oxidized and bruised apple (Rapp and Mandery, 1986), but its presence in small amounts is essential to achieve color stability in red wines thanks to the formation of stable and intensely colored anthocyanin-tannin polymers (Es-Safi et al., 1999, 2002). On the other hand, uncontrolled contact with air induces formation of other aldehydes such as octanal, nonanal, decanal, and methional, that have a central role in the formation of some oxidized and cooked vegetable nuances (Escudero et al., 2000, 2002; Culleré et al., 2007).

The criteria to define the most optimal degree of oxygen exposure of a given wine remain somewhat difficult to describe. The outcomes of oxygen exposure are highly wine dependent (Singleton, 1987; Ugliano, 2013), being in large part linked to wine phenolic composition (Wirth et al., 2010, 2012), presence of exogenous and endogenous antioxidants in wines and musts (Godden et al., 2005; Ugliano et al., 2010; Motta et al., 2014), and aroma composition of the wine, in particular regarding its volatile sulfur compounds profile (Ugliano et al., 2011; Ugliano, 2013). Therefore, for a given amount of oxygen consumed by the wine during a defined period of time, certain wines can show positive outcomes whereas in other cases negative consequences can be observed, e.g., loss of fresh fruity aromas. In this sense, the concept of wine oxygen demand, namely the most suitable degree of oxygen exposure of a given wine, has been recently discussed (Ugliano, 2013; Carrascon et al., 2015; Ferreira et al., 2015; Marrufo-Curtido et al., 2017) but remains difficult to rationalize.

Nebbiolo grapes are used in the production of premium Italian wines suitable for long bottle aging, such as the famous Barolo and Barbaresco denominations of origin from the Piedmont region (northwest Italy). Studies on the oxidizable compounds of Nebbiolo grapes and wines indicated a peculiar phenolic composition with relatively high contents of peonidin-3-glucoside and cyanidin-3-glucoside (Rio Segade et al., 2015; Torchio et al., 2016). Based on these observations, it was suggested that musts and wines obtained from Nebbiolo grapes could be particularly sensitive to oxidation, because of the greater oxidability of these di-substituted anthocyanins (Cheynier et al., 1994; Sarni et al., 1995; Cagnasso et al., 2008). Accordingly, specific winemaking practices should be adopted in the production of wines from grapes with these characteristics aimed at stabilizing the reactivity of these compounds, including those promoting oxidation-mediated condensation of anthocyanins with tannins leading to colored pigments (Fulcrand et al., 1996; Saucier et al., 1997a,b).

Gerbi et al. (2006) investigated the influence of micro-oxygenation on the composition and quality of wines from Nebbiolo grapes, indicating a major influence on color characteristics. Despite the critical role of oxidative phenomena in Nebbiolo winemaking, to our knowledge no other data is available on the influence of oxygen exposure on Barolo and Barbaresco wines under oxygen exposure levels similar to those occurring during common cellar handling, which are typically characterized by higher oxygen compared to micro-oxygenation.

In this study, we have investigated the influence of repeated consumption of relatively large (e.g., 7 mg/L) doses of oxygen on the phenolic and volatile composition of two Barolo and two Barbaresco area wines, providing a first insight in the behavior of these wines during early stage of oxidation and storage.

## Materials and methods

### Chemicals

For GC-MS analysis, dichloromethane, anhydrous sodium sulfate and ammonium sulfate used during sample preparation were purchased from Scharlab Italia S.r.l. (Riozzo di Cerro al Lambro, Italy). Sulphuric acid, sodium hydroxide (6N) and hydrochloric acid 6M were obtained from Fluka (Steinheim, Germany). The derivatizing agent PFBHA [o-(2,3,4,5,6-pentafluorobenzyl) hydroxylamine hydrochloride], the reference substances 3-(methylthio)-propanal (methional), *t*-2-nonenal, phenylacetaldehyde, *t*-2-hexenal, *t*-2-octenal, and the internal standard 2-methylpenthanal were supplied by Sigma-Aldrich (Steinheim, Germany).

For HPLC aldehyde determinations, all reagents and solvents, including dinitro phenyl hydrazine (DNPH), sulphuric acid, acetonitrile, perchloric acid, phosphate buffer were purchased from Sigma-Aldrich (Steinheim, Germany).

All other reagents and solvents used for analysis were obtained at the maximum grade of purity available from Sigma-Aldrich (Steinheim, Germany).

### Experimental plan and wines treatment

**Figure 1:**
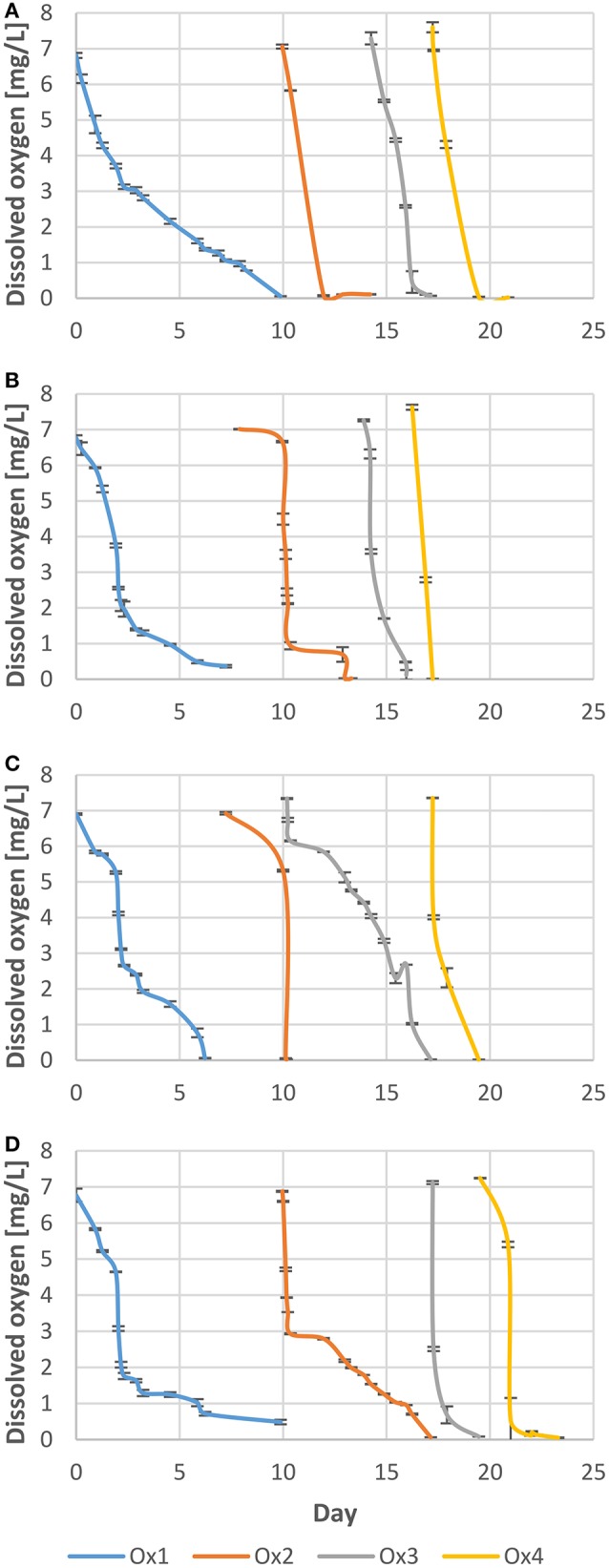
Oxygen consumption kinetics of the four wines considered (Ne1 to Ne4 in quadrants **A–D**, respectively). The four oxygenation levels are reported as Ox1 to Ox4 curves.

Four Nebbiolo wines from 2013 vintage were used during the experiment: two of them come from the Barbaresco production area (Ne1 and Ne2 wines), while the remaining two come from Barolo production area (Ne3 and Ne4 wines). For each sample, 25 L of wine were used. The wines were produced using exclusively Nebbiolo grapes and taken directly from the cellars. Prior to the experiment, the free sulfur dioxide (SO_2_) content was adjusted in all wines at 25 ± 2 mg/L, with total SO_2_ values (*n* = 3) being 44 ± 2 mg/L, 60 ± 3 mg/L, 37 ± 2 mg/L, 45 ± 4 mg/L for Ne1, Ne2, Ne3, and Ne4, respectively. Wines were analyzed at the beginning of the experiment (T0) and then enriched with oxygen by stirring in air until a concentration of dissolved oxygen of 7.0 ± 0.5 mg/L was achieved (Figure 1). Then, the wines were poured in 2-L glass bottles, with dissolved oxygen constantly monitored. For each bottle separately, when dissolved oxygen reached a value lower than 0.5 mg/L, the oxidation experiment was either stopped, or another cycle of oxygen enrichment/consumption was initiated, up to a maximum of four cycles. Four degrees of oxidation were therefore obtained (Ox1, Ox2, Ox3, and Ox4 samples), corresponding to about 7, 14, 21, and 28 mg/L of consumed oxygen respectively to simulate a winery condition and forced oxidation levels (Castellari et al., 2004; Picariello et al., 2018). Afterwards, samples were stored for 300 days in the absence of oxygen, to investigate their evolution following the oxidation cycles. All experiments were carried out in duplicate in glass bottles sealed with air-tight crown caps. Analyses were carried out after 60 days (T60) and 300 days (T300). Bottles were stored at 20 ± 1°C, a condition considered halfway between cellar condition and typical domestic wine conservation (Arapitsas et al., 2014).

### Wine analysis

Physicochemical parameters were measured before oxygenation (T0). Titratable acidity, pH, and ethanol data were obtained using OIV official methods (OIV, 2016). Organic acids (tartaric, malic, and lactic acid) and residual sugars (glucose and fructose) were quantified respectively by DAD-HPLC and RI-HPLC (Giordano et al., 2009). Copper and iron have been quantified by atomic absorption spectrometry (OIV, 2016).

### Dissolved oxygen

The dissolved oxygen content was assessed by a portable analyzer (NomaSense O2 P300, Nomacorc, Zebulon, NC, USA) equipped with Dissolved Oxygen Sensors luminescence LDO as well as temperature and pressure sensors for correction to standard values. Dissolved oxygen has been expressed in mg/L.

### Color analysis and polyphenolic profile of wines

All spectrophotometric analyses were carried out using a UV-1800 spectrophotometer (Shimazdu Corporation, Kyoto, Japan). Wine color has been studied by evaluating the color intensity expressed as A_420_+A_520_+A_620_ and the hue expressed as A_420_/A_520_ (OIV, 2016). The trichromatic parameters in CIEL^*^a^*^b^*^ color space were also calculated using the algorithm proposed by Piracci (OIV, 2016). Moreover, the colored compounds were also fractionated following the method proposed by Glories (1984) and simplified by Di Stefano and Cravero (1989). Consequently the 520 nm absorbing pigments in wine were fractionated depending on their reaction with SO_2_. Thus, the contribution to wine color was divided into non-bleachable structure (dTAT%), sensitive to discoloration pigments (dAT%), and free anthocyanins (dAl%). The copigmentation color indexes (copigmented anthocyanins, free anthocyanins and polymeric pigments fractions) were also determined according to the method proposed by Boulton (1996).

Phenolics in wine were evaluated by spectrophometric methods. Total polyphenol content was determined according to Singleton and Rossi (1965). Flavan-3-ols were evaluated by the proanthocyanidins index, using the Bate-Smith reaction by heating wine in acidic medium (Glories, 1984; Di Stefano et al., 1989), reading absorbance at 532 nm on 10 mm path length cuvette; flavans were also characterized by their reactivity toward vanillin in accordance with the method proposed by Di Stefano and Cravero (1991). Briefly, the total anthocyanin content was determined by measuring the maximum absorbance at 536-540 nm after dilution with ethanol:water:hydrochloric acid 37% in the proportion 70:30:1 *v/v* (hydrochloric ethanol), while anthocyanins in their monomeric form were determined by retaining polymeric anthocyanins on a solid phase extraction (SPE) cartridge using polyvinylpolypyrrolidone (PVPP) as a stationary phase and hydrochloric ethanol as elution solvent. The spectrophotometric measurement of the extract thus obtained was carried out at a wavelength of 540 nm on 10 mm path length cuvette (Di Stefano et al., 1989). The relative standard deviations of phenolic compound determination, based on repeated analyses (*n* = 20) of ten sample extracts, were found in a previous work of 1.14, 2.80, and 1.74% for total anthocyanins index, vanillin assay, and proanthocyanidins determinations, respectively (Torchio et al., 2010).

#### Volatile aldehydes by GC-MS

Hexenal, *t*-2-octenal, *t*-2-nonenal, phenylacetaldehyde, and methional, were derivatised by means of PFBHA. Adducts were separated and detected by gaschromathography-mass spectrometry (GC-MS). The analytical method is based on a Micro Liquid-Liquid Extraction (MLLE) strategy described by previous studies (Bao et al., 1998; Ortega et al., 2001; Ferreira et al., 2006; Beránek and Kubátová, 2008; Carrillo et al., 2011) Samples were prepared as follows: wine pH was adjusted to 4.00 using a 6 mol/L NaOH solution prior to derivatization procedure. Two hundred microliter of internal standard (2-methylpentanal, 1.32 mg/mL), 2 mL of ultra-pure water and 500 μL of PFBHA HCl aqueous solution 3 mg/L were added in a N_2_ saturated tube containing 4 mL of wine.

The tubes were tightly sealed and placed in a thermostatic bath at 50°C for 105 min. At the end of the derivatization process, samples were cooled at room temperature, then 500 μL of H_2_SO_4_ 10N, 2.5 g of ammonium sulfate, and 250 μL Dichloromethane (DCM) were separately added to each tube for the liquid-liquid extraction of the derivatives. Samples were shaken in an orbital agitator at minimum speed to avoid emulsion formation. After 1.5 h the tubes were centrifuged at 4,000 rpm for 10 min at 10°C to allow the organic phase to separate. The aqueous layer was eliminated by using a Pasteur glass pipette and the organic fraction was put into 2-mL vials including a 250 μL micro volume vial insert. Samples were stored at −18°C until analysis (not longer than 15 days). Before analysis, anhydrous sodium sulfate was added to the sample to remove water.

The GC-MS analysis was performed on an Agilent 6890 Series gas chromatograph equipped with a single quadrupole Agilent 5973N detector (Agilent Technologies, Santa Clara, CA, US). Samples (1 μL of DCM extract) were injected at 250°C in splitless mode. The column was a Stabilwax-MS, 30 m × 0.25 mm × 0.25 μm stationary phase thickness (Restek Italia, Cernusco sul Naviglio, Italy). The oven temperature was maintained at 45°C for 2 min, then it was raised to 60°C with a rate of 30°C/min, from 60 to 230°C at a rate of 2°C/min, and then the temperature was kept at 230°C for 20 min. The carrier gas was helium with a constant flow of 1 mL/min. The GC transfer line was kept at 230°C. The electron energy was set at 70 eV and the source temperature was 250°C. The quadrupole temperature was 230°C. Single ion monitoring (SIM) was used for the quantitation of studied compounds. Dwell time was 100 μs for each ion. Selected ions for quantification and identification were 253 and 181 *m/z* for 2-methylpentanal (used as internal standard), 252 and 181 *m/z* for methional, 250 and 181 *m/z* for t-2-nonenal, 117, 91, and 181 *m/z* for phenylacetaldehyde, 230 and 181 *m/z* for t-2-octenal, 293, 250 and 181 *m/z* for t-2-hexenal. The identification of aldehydes was carried out by comparing the retention index of the compounds of interest with those of a pure derivatized standard and analyzed under the same conditions. Quantitative data were obtained by interpolating the peaks areas relative to the calibration graphs constructed by the analysis of synthetic wines containing known amounts of the analytes. Sample chromatograms are provided in Figures S1–S5. Each sample was analyzed in duplicate.

#### Major aldehydes by HPLC

Acetaldehyde, glyceraldehyde and glyoxylic acid were analyzed by HPLC. Analysis were carried out after derivatization with DNPH as proposed by Nishikawa and Sakai (1995), adapted by Elias et al. (2008), and modified as follows: 1 mL of wine was acidified with 200 μL of 25% concentrated sulphuric acid. To the solution was added 1 mL of DNPH in acetonitrile prepared by diluting 1 mL of DNPH (saturated solution in acetonitrile) in 100 mL of acidified acetonitrile with 4 mL of 70% perchloric acid. The solution thus prepared is stored at 4°C. Subsequently, the sample was placed in a 4-mL vial, flushed with nitrogen, closed with a screw-cap, slightly agitated, and placed in the dark at 25°C for 3 h. Each sample was supplemented with 2 mL of 25 mM phosphate buffer at pH 2.2. Samples were filtered on a 0.22-μm filter and analyzed by HPLC.

The analyses were carried out on an Agilent 1100 HPLC equipped with a LiChrosper 250 × 4 mm (5 μm particle size) column. The flow rate was 0.4 mL/min, the injection volume was 25 μL and the signal was acquired at 365 nm using a DAD detector. Phase A was 1% acetonitrile in phosphate buffer 25 mM, pH 2.20, phase B was acetonitrile. The following gradient was established: at 0 min 75% A and 25% B, after 10 min 50% A and 50% B, after 30 min 75% A and 25% B, after 45 min 25% A and 75% B. A sample chromatogram is provided in Figure S6.

#### Statistical analysis

Data from chemical analyses were elaborated by the Analysis of Variance (ANOVA) using the software SPSS Windows version 19.0 (IBM Corporation, Armonk, NY, USA). The Tukey-b *post-hoc* test (*p* < 0.05) was used when one-way analysis of variance (ANOVA) showed significant differences in the results obtained. The Principal Component Analysis (PCA) was carried out using XLStat Statistical Software (XLSTAT 2017: Data Analysis and Statistical Solution for Microsoft Excel. Addinsoft, Paris, France, 2017).

## Results

The Nebbiolo wines presented a different initial composition. In the supplementary material (Tables S1-S13) are reported, separately for each wine, the chemical-physical parameters detected during the experiment.

### General physicochemical composition of the wines

Main chemical characteristics of the wines used in this study are summarized in Table S1. The alcoholic fermentation was successfully completed for all wines. Barbaresco (Ne1 and Ne2) wines had a higher ethanol level with respect to Barolo wines (Ne3, Ne4). The pH of all the wines studied ranged between 3.39 and 3.55, and titratable acidity (expressed as tartaric acid) ranged from 4.96 to 5.74 g/L. The malolactic fermentation was completed in all samples and only negligible levels of malic acid were measured. Lactic acid content ranged from 1.01 to 1.79 g/L. The metals content was low in all wines, with a slightly higher copper concentration for Ne3, which contained 0.16 mg/L of Cu.

### Color parameters and polyphenolic content

The Nebbiolo wines employed in the trial were different for most color and phenolic parameters that were analyzed (Tables S2–S9) at beginning of the experiment (T0). In particular, color intensity varied from 4.95 (Ne3) to 8.12 a.u. (Ne4), while color hue from 0.74 (Ne4) to 0.94 (Ne3). Since the wines had similar content of free SO_2_, differences in wine color were probably due to content of total and free antocyanins, which respectively varied from 118 (Ne3) to 179 mg/L (Ne4) for the former, and from 41 (Ne1) to 73 mg/L (Ne2) for the latter. In addition, the dTAT% parameter (pigments not bleachable by SO_2_) varied from 19.2 to 28.3% (Ne2 and Ne1), and the pH values were found from 3.39 to 3.55 (Ne1-Ne4 and Ne3). The dTAT% values represent an index of the degree of wine color evolution and their variation was associated with that of polymeric pigments.

The average content of total polyphenols (Folin-Ciocalteu assay) in the wines varied from 2,833 (Ne1) to 3,635 mg/L (Ne4), while condensed tannins (proanthocyanidins, PC) from 3,595 (Ne3) to 4,171 mg/L (Ne4), and flavans reactive with vanillin (flavans with a low molecular weight, FRV) from 1,931 (Ne1) to 2,994 mg/L (Ne4).

All wines were characterized by a low content of anthocyanins and a high content of polyphenols, a peculiarity of the wines obtained from the grapes of the Nebbiolo cultivar.

Tables 1, 2 report the parameters that describe the average wines chromatic characteristics. The factors studied were the storage time (Table 1) and the oxygen dose supplied (Table 2). Significant differences among the treatments with different oxygen intakes in color parameters (a^*^, b^*^, and L^*^ values, hue and intensity) were observed both after 60 and 300 days of storage (T60 and T300, respectively). Significant differences for color intensity between each treatments combination were observed, except after 300 days of storage in Ne1 and Ne2 wines: in the Ne1 wines the doses Ox2, Ox3, and Ox4, and in the Ne2 the doses Ox2 and Ox3 were not significantly different among them.

**Table 1:** Color characteristics of wines during the trial referred to each oxygenation level.

**Oxygenation level**	**Storage time/significance**	L^*^	a^*^	b^*^	**Color hue**	**Color intensity^1^**	**dTAT [%]**	**dAL [%]**	**dAT [%]**	**Copigmentation fraction [%]**	**Free anthocyanins fraction [%]**	**Polymeric pigments fraction [%]**
Ox1	T0	25.4 a	53.8 a	46.2 b	0.85 c	6.3 c	22.4 c	26.3 a	51.3 b	18.1 a	37.3	44.6 b
	T60	15.1 b	43.3 b	30.8 c	1.12 a	7.6 a	25.9 b	17.4 c	56.7 a	13.6 b	38.4	48.0 b
	T300	25.6 a	53.9 a	48.1 a	0.91 b	6.5 b	32.0 a	22.0 b	46.1 c	8.5 c	34.5	57.0 a
	Sign.^a^	^***^	^***^	^***^	^***^	^***^	^***^	^***^	^***^	^***^	^*^	^***^
	Sign (Time × wine)^b^	^***^	^***^	^***^	^***^	^***^	^***^	ns	^**^	ns	^*^	ns
Ox2	T0	25.4 a	53.8 a	46.2 b	0.86 c	6.3 c	22.4 c	26.3 a	51.3 b	18.1 a	37.3b	44.6 c
	T60	14.2 c	42.9 c	30.0 c	1.10 a	7.9 a	27.1 b	16.0 c	57.0 a	11.6 b	39.8a	48.6 b
	T300	24.3 b	53.3 b	46.9 a	0.90 b	6.7 b	34.3 a	20.3 b	45.4 c	11.2 b	31.6c	57.2 a
	Sign.^a^	^***^	^***^	^***^	^***^	^***^	^***^	^***^	^***^	^***^	^***^	^***^
	Sign (Time × wine)^b^	^***^	^***^	^***^	^***^	^***^	ns	^*^	ns	ns	^*^	^***^
Ox3	T0	25.4 a	53.8 a	46.2 a	0.86 c	6.3 c	22.4 c	26.3 a	51.3 b	18.1 a	37.3 b	44.6 c
	T60	12.9 c	42.0 c	28.6 c	1.08 a	8.2 a	27.9 b	14.7 c	57.4 a	11.1 b	39.4 a	49.4 b
	T300	23.3 b	52.7 b	45.9 b	0.90 b	6.9 b	37.6 a	18.6 b	43.8 c	8.6 c	33.3 c	58.0 a
	Sign.^a^	^***^	^***^	^***^	^***^	^***^	^***^	^***^	^***^	^***^	^***^	^***^
	Sign (Time × wine)^b^	^***^	^***^	^***^	^***^	^***^	^**^	^**^	ns	^***^	^***^	^***^
Ox4	T0	25.4 a	53.8 a	46.2 a	0.86 c	6.3 c	22.4 c	26.3 a	51.3 b	18.1 a	37.3 a	44.6 c
	T60	12.3 c	41.4 c	27.8 c	1.06 a	8.3 a	28.3 b	13.2 c	58.2 a	11.3 b	38.8 a	49.9 b
	T300	22.5 b	52.1 b	45.0 b	0.90 b	7.1 b	38.8 a	16.6 b	44.7 c	8.2 c	32.2 b	59.6 a
	Sign^a^	^***^	^***^	^***^	^***^	^***^	^***^	^***^	^***^	^***^	^***^	^***^
	Sign (Time × wine)^b^	^***^	^***^	^***^	^***^	^***^	^*^	^**^	ns	ns	^*^	^***^

Values expressed as average (n = 8). Different Latin letters within the same column and oxygenation level indicate significant differences among storage times (T0, T60, T300) at p < 0.05 (Tukey-b test).

*, **, *** *, and ns indicate significance at p < 0.05, 0.01, 0.001, and not significant, respectively, among storage times within the same column and oxygenation level (^a^), or of the interaction between “storage time” and “wines” in a two-way ANOVA (^b^)*.

1 *Expressed as A.U.—optical path 10 mm*.

**Table 2:** Color characteristics of wines during the trial referred to each storage time.

**Storage time**	**Oxygenation level**	L^*^	a^*^	b^*^	**Color hue**	**Color intensity^1^**	**dTAT [%]**	**dAL [%]**	**dAT [%]**	**Copigmentation fraction [%]**	**Free anthocyanins fraction [%]**	**Polymeric pigments fraction [%]**
T60	Ox1	15.1 a	43.3 a	30.8 a	1.12 a	7.6 d	25.9	17.4 a	56.7	13.6 a	38.4	48.0 b
	Ox2	14.2 b	42.9 b	30.0 b	1.10 b	7.9 c	27.1	16.0 ab	57.0	11.6 ab	39.8	48.6 b
	Ox3	12.9 c	42.0 c	28.6 c	1.08 c	8.2 b	27.9	14.7 b	57.4	11.1 b	39.4	49.4 a
	Ox4	12.3 d	41.4 d	27.8 d	1.06 d	8.3 a	28.3	13.2 c	58.2	11.3 b	38.8	49.9 a
	Sign.^a^	^***^	^***^	^***^	^***^	^***^	ns	^***^	ns	^*^	ns	^***^
	Sign.^b^ (Ox × wine)	^***^	^***^	^***^	^***^	^***^	ns	^**^	ns	ns	ns	ns
T300	Ox1	25.6 a	53.9 a	48.1 a	0.894 d	6.5 d	32.0 b	22.0 a	46.1	8.5 b	34.5	57.0
	Ox2	24.6 b	53.4 b	47.1 b	0.898 c	6.7 c	34.0 b	20.6 a	45.4	11.5 a	31.5	57.0
	Ox3	23.3 c	52.7 c	45.9 c	0.904 b	6.9 b	37.6 a	18.6 b	43.8	8.6 b	33.3	58.0
	Ox4	22.5 d	52.1 d	45.0 d	0.912 a	7.1 a	38.8 a	16.6 c	44.7	8.2 b	32.2	59.6
	Sign^a^	^***^	^***^	^***^	^***^	^***^	^***^	^***^	ns	^*^	ns	ns
	Sign.^b^ (Ox × wine)	^***^	^***^	^***^	^***^	^***^	^*^	^*^	ns	ns	ns	ns

Values expressed as average (n = 8). Different Latin letters within the same column and storage time indicate significant differences among oxygenation levels at p < 0.05 (Tukey-b test).

*, **, *** *, and ns indicate significance at p < 0.05, 0.01, 0.001, and not significant, respectively, among oxygenation levels within the same column and storage time (^a^), or of the interaction between “oxygenation level” and “wines” in a two-way ANOVA (^b^)*.

1 *Expressed as A.U.—optical path 10 mm*.

With regard to color evolution over time (Table 1), during the first 60 days of storage an increase in color intensity was observed for each degree of oxygenation. Then, these values tended to decrease over time, but always remaining above the initial value. The hue increased during the first 2 months of storage showing an increase in yellow color, and subsequently fell, indicating an increase in red color, an uncommon behavior which may have been caused by the SO_2_ combination with anthocyanins at T0. Regarding the CIEL^*^a^*^b^*^ parameters, a significant decrease of a^*^, b^*^, and L^*^ values was observed in the early stages of storage that was followed by an increase after 300 days of storage. After 300 days of storage (Table 2), significant differences for L^*^, a^*^, and b^*^ parameters and hue were observed among treatments with different degree of oxidation: the three chromatic coordinates progressively decreased, while hue increased as the dose of oxygen increased.

The effect of oxygen intakes was not the same for all wines (significant interactions between oxygen and wine): after 300 days of storage it was observed, that only in the Ne3 and Ne4 wines at each increase of the dose of oxygen corresponded a significant increase in color intensity. On the contrary, in Ne1 wine the increase was significant only passing from Ox1 to Ox2, while in Ne2 wine, for the same parameter, Ox2 and Ox3 were not significantly different from each other.

Regarding other parameters describing wine color characteristics, monomeric anthocyanins (dAl%) and SO_2_-bleachable pigments (dAT%) percentage contribution to red color (measured as absorbance at 520 nm) decreased during wine storage, as well as the copigmentation effect. On the other hand, the percentage contribution of SO_2_ non-bleachable (dTAT%) pigments and polymeric structures to red color increased (Table 1).

After 60 days of storage, no significant differences were observed between the samples added of different doses of oxygen with regard to dTAT% and dAT%. On the contrary, a significant reduction of dAl% with increasing the dose of oxygen was noticed. After 300 days of storage, significant differences were observed for dTAT% and dAl%. The dTAT% and dAl% respectively increased and decreased as the dose of oxygen increased, but not all treatments resulted significantly different from each other.

Also, for the dTAT% and dAl% parameters there were differences in behavior between wines (significant interactions between oxygen and wine). Considering the different wines separately (Tables S2-S5), after 300 days of storage the effect of increasing oxygen supplies resulted only significant in 2 wines out of 4 for dTAT% and in 3 wines out of 4 for dAl%.

Finally, the effect of oxygenation on copigmentation and on the polymeric pigments was modest, although statistically significant in some cases. The copigmentation fraction (%) slightly decreased, whereas the polymeric pigment fraction slightly increased as the dose of oxygen increased.

During aging (Table 3), a progressive decrease of the content of total and free anthocyanins was observed. The differences between the wines added of different doses of oxygen (Table 4) were significant: by increasing the amount of supplied oxygen the content of anthocyanins decreased although the magnitude of this phenomenon was modest and did not concern all the wines.

**Table 3:** Phenolic profile of wines during the trial referred to each oxygenation level.

**Oxygenation level**	**Storage time**	**Total anthocyanin index^1^**	**Total flavonoids index^2^**	**Monomeric anthocyanins index^1^**	**Total polyphenols index^2^**	**Proanthocyanidins index (PC)^3^**	**Vanillin assay (FRV)^2^**	**FRV/PC ratio**
Ox1	T0	147.3 a	2863 a	55.6 a	3201 b	3935	2475 a	0.63 a
	T60	135.0 b	2836 a	32.8 b	3552 a	3932	2532 a	0.64 a
	T300	102.3 c	2715 b	19.1 c	3548 a	3898	2206 b	0.56 b
	Sign.^a^	^***^	^***^	^***^	^***^	ns	^***^	^***^
	Sign (Time × wine)^b^	^***^	ns	^***^	^**^	^***^	ns	ns
Ox2	T0	147.3 a	2863 a	55.6 a	3201 b	3935	2475 a	0.63 a
	T60	130.1 b	2813 b	29.4 b	3574 a	3864	2538 a	0.65 a
	T300	99.9 c	2678 c	15.8 c	3493 a	3930	2106 b	0.53 b
	Sign.^a^	^***^	^***^	^***^	^***^	ns	^***^	^***^
	Sign (Time × wine)^b^	^***^	ns	^***^	^**^	^***^	ns	ns
Ox3	T0	147.3 a	2864 a	55.6 a	3201 b	3935	2475 a	0.63 a
	T60	128.4 b	2837 a	26.6 b	3609 a	3862	2507 a	0.64 a
	T300	98.6 c	2683 b	14.2 c	3562 a	3923	2133 b	0.58 b
	Sign.^a^	^***^	^***^	^***^	^***^	ns	^***^	^***^
	Sign (Time × wine)^b^	^***^	ns	^***^	^***^	^**^	ns	ns
Ox4	T0	147.3 a	2864 a	55.6 a	3201 b	3935 a	2475 a	0.63 b
	T60	124.5 b	2758 b	23.7 b	3497 a	3732 b	2524 a	0.67 a
	T300	97.9 c	2655 c	13.5 c	3569 a	3880 ab	2133 b	0.54 c
	Sign^a^	^***^	^***^	^***^	^***^	^*^	^***^	^***^
	Sign (Time × wine)^b^	^***^	ns	^***^	^**^	^*^	ns	ns

Values expressed as average (n = 8). Different Latin letters within the same column and oxygenation level indicate significant differences among storage times (T0, T60, T300) at p < 0.05 (Tukey-b test).

*, **, *** *, and ns indicate significance at p < 0.05, 0.01, 0.001, and not significant, respectively, among storage times within the same column and oxygenation level (^a^), or of the interaction between “storage time” and “wine” in a two-way ANOVA (^b^)*.

1 *Expressed as [mg malvidin-3-glucoside chloride/L]*.

2 *Expressesd as [mg (+)-catechin/L]*.

3 *Expressed as [mg cyanidin chloride/L]*.

**Table 4:** Phenolic profile of wines during the trial referred to each storage time.

**Storage time**	**Oxygenation level**	**Total anthocyanin index^1^**	**Total flavonoids index^2^**	**Monomeric anthocyanins index^1^**	**Total polyphenols index^2^**	**Proanthocyanidins index (PC)^3^**	**Vanillin assay (FRV)^2^**	**FRV/PC ratio**
T60	Ox1	135 a	2836 a	32.8 a	3552	3932 a	2532	0.64 b
	Ox2	130 b	2814 ab	29.4 b	3574	3864 a	2538	0.65 ab
	Ox3	128 b	2837 a	26.6 c	3609	3862 a	2507	0.64 b
	Ox4	125 c	2758 b	23.7 d	3497	3732 b	2524	0.67 a
	Sig.	^***^	^*^	^***^	ns	^**^	ns	^**^
	Sign^c^ (Ox × wine)	^**^	ns	^**^	ns	ns	^**^	^*^
T300	Ox1	102.3 a	2715 a	19.1 a	3548 ab	3898	2206 a	0.56 a
	Ox2	100.4 b	2678 bc	16.3 b	3505 b	3927	2124 b	0.54 b
	Ox3	98.6 c	2683 b	14.2 c	3562 a	3923	2133 ab	0.54 ab
	Ox4	97.9 c	2655 d	13.5 c	3569 a	3880	2133 ab	0.55 ab
	Sig.	^***^	^***^	^***^	^*^	ns	^*^	^*^
	Sign^c^ (Ox × wine)	^***^	^**^	^*^	^**^	ns	^*^	ns

Values expressed as average (n = 8). Different Latin letters within the same column and storage time indicate significant differences among oxygenation levels at p < 0.05 (Tukey-b test).

*, **, *** *, and ns indicate significance at p < 0.05, 0.01, 0.001, and not significant, respectively, among oxygenation levels within the same column and storage time (^a^), or of the interaction between “oxygenation level” and “wines” in a two-way ANOVA (^b^)*.

1 *Expressed as [mg malvidin-3-glucoside chloride/L]*.

2 *Expressesd as [mg (+)-catechin/L]*.

3 *Expressed as [mg cyanidin chloride/L]*.

A moderate increase over time (Table 3) in the total polyphenol index was observed for all wines, especially during the first storage time (between T0 and T60). On the contrary, the amount of low molecular flavans (FRV) decreased after 300 days of storage, following a slight increase after 60 days of storage. A similar behavior was observed for the FRV/PC ratio, which showed an increase during the first months of storage (T60), followed by a general decrease until 300 days of storage. The proanthocyanidins index decreased after 60 days of storage, then it increased during aging: oxidation and depolymerization processes of polymeric phenols can explain this complex behavior. However, the differences were statistically significant only within Ox4 (Table 3).

On the whole, the different oxygen supplies did not modify the wines polyphenolic composition; in some cases, there were significant differences between treatments, but they were not linked to the dose of oxygen.

### Aldehydic compounds

The concentration of glyoxylic acid significantly increased over time in all wines, regardless of the supplied oxygen (Tables 5, 6). At the same time glyceraldehyde concentration increased significantly only after 10 months of storage (T300). On the other hand, acetaldehyde dramatically increased at 60 days of storage and the increases were directly associated with the amount of oxygen added.

**Table 5:** Aldeyde profile of wines during the trial referred to each oxygenation level.

**Oxygenation level**	**Storage time**	**Glyceraldehyde^1^**	**Glyoxylic acid^1^**	**Acetaldehyde^1^**	***t*-2-Hexenal^2^**	***t*-2-Octenal^2^**	**Methional^2^**	***t*-2-Nonenal^2^**	**Phenylacetaldehyde^2^**
Ox1	T0	17.8 b	0.78 c	4.10 a	1.2 b	0.4 ab	4.8 b	4.5 b	55.9 ab
	T60	17.3 b	1.16 b	3.86 a	0.8 b	0.3 b	3.6 c	2.3 c	32.9 b
	T300	25.2 a	1.76 a	2.31 b	2.1 a	0.5 a	7.4 a	8.7 a	128.6 a
	Sign.^a^	^***^	^***^	^***^	^***^	^*^	^***^	^***^	^*^
	Sign (Time × wine)^b^	^***^	^***^	^***^	ns	ns	^***^	^***^	ns
Ox2	T0	17.8 b	0.78 c	4.11 b	1.2 ab	0.4	4.8 ab	4.5 b	55.9 ab
	T60	16.7c	1.32 b	5.60 a	0.8 b	0.3	3.8 b	2.3 c	30.9 b
	T300	24.9 a	1.86 a	3.63 c	1.5 a	0.4	9.0 a	6.3 a	109.8 a
	Sign.^a^	^***^	^***^	^***^	^**^	ns	^*^	^***^	^**^
	Sign (Time × wine)^b^	^***^	^**^	^***^	ns	ns	ns	^**^	ns
Ox3	T0	17.8 b	0.78 c	4.11 b	1.2 b	0.4	4.8 b	4.5 b	55.9 b
	T60	16.8 c	1.51 b	7.41 a	0.8 c	0.3	4.0 b	2.3 c	30.4 c
	T300	24.7 a	1.98 a	3.55 c	2.0 a	0.5	7.1 a	7.7 a	132.0 a
	Sign.^a^	^***^	^***^	^***^	^**^	ns	^***^	^***^	^***^
	Sign (Time × wine)^b^	^***^	^**^	^***^	ns	^*^	^***^	^***^	^***^
Ox4	T0	17.8 b	0.78 c	4.11 b	1.2 ab	0.4 ab	4.8 b	4.5 b	55.9 b
	T60	17.4 b	1.78 b	9.50 a	0.9 b	0.3 b	4.1 b	2.2 c	33.3 b
	T300	24.9 a	2.21 a	3.94 b	1.8 a	0.4 a	6.4 a	5.9 a	112.8 a
	Sign.^a^	^***^	^***^	^***^	^*^	^*^	^***^	^***^	^***^
	Sign (Time × wine)^b^	^***^	^***^	^***^	ns	^*^	^*^	^***^	^**^

Values expressed as average (n = 8). Different Latin letters within the same column and oxygenation level indicate significant differences among storage times (T0, T60, T300) at p < 0.05 (Tukey-b test).

*, **, *** *, and ns indicate significance at p < 0.05, 0.01, 0.001, and not significant, respectively, among storage times within the same column and oxygenation level (^a^), or of the interaction between “storage time” and “wines” in a two-way ANOVA (^b^)*.

1 *Expressed as mg/L*.

2 *Expressed as μg/L*.

**Table 6:** Aldeyde profile of wines during the trial referred to each storage time.

**Storage time**	**Oxygenation level**	**Glyceraldehyde^1^**	**Glyoxylic acid^1^**	**Acetaldehyde^1^**	***t*-2-Hexenal^2^**	***t*-2-Octenal^2^**	**Methional^2^**	***t*-2-Nonenal^2^**	**Phenylacetaldehyde^2^**
T60	Ox1	17.3 a	1.16 a	3.86 a	0.80	0.25	3.58 a	2.25	32.9
	Ox2	16.7 b	1.33 b	5.60 b	0.84	0.26	3.75 ab	2.27	30.9
	Ox3	16.8 b	1.51 c	7.41 c	0.77	0.33	4.03 ab	2.27	30.4
	Ox4	17.4 a	1.78 d	9.50 d	0.86	0.25	4.07 b	2.19	33.3
	Sig.	^**^	^***^	^***^	ns	ns	^*^	ns	ns
	Sign^c^ (Ox × wine)	^***^	^***^	^***^	ns	ns	ns	ns	^*^
T300	Ox1	25.2	1.76 c	2.31 c	2.15	0.47	7.38	8.73 a	128.6
	Ox2	25.2	1.83 bc	3.24 b	1.60	0.42	9.15	6.92 bc	117.4
	Ox3	24.7	1.98 b	3.56 ab	1.96	0.54	7.13	7.67 ab	132.0
	Ox4	24.9	2.21 a	3.94 a	1.82	0.43	6.36	5.85 c	112.8
	Sig.	ns	^***^	^***^	ns	ns	ns	^***^	ns
	Sign^c^ (Ox × wine)	^***^	ns	^***^	ns	ns	ns	^**^	ns

Values expressed as average (n = 8). Different Latin letters within the same column and storage time indicate significant differences among oxygenation levels at p < 0.05 (Tukey-b test).

*, **, *** *, and ns indicate significance at p < 0.05, 0.01, 0.001, and not significant, respectively, among oxygenation levels within the same column and storage time (^a^), or of the interaction between “oxygenation level” and “wines” in a two-way ANOVA (^b^)*.

1 *Expressed as mg/L*.

2 *Expressed as μg/L*.

From 2 until 10 months of storage (Table 5), acetaldehyde levels decreased and reached the minimum values after 300 days of storage. This trend was observed in all wines except for the less exposed to oxygen (Ox1) where no accumulation was highlighted. Concentrations above 10 mg/L at T60 were evidenced in Ne3 and Ne4 wines subjected to the highest oxygenation level (Ox4) (Tables S10–S13).

As in the case of acetaldehyde, glyoxylic acid content increased concurrently with oxygen intake, but after 300 days of storage only in one wine out of four (Ne1) significant differences between the wines with different doses of oxygen were noticed.

With regards to minor volatile aldehydes (t-2-hexenal, t-2-octenal, methional, t-2-nonenal, phenylacetaldehyde) a slight decrease was observed until 60 days of storage, then aldehydes concentration tends to increase overall and significant differences between T60 and T300 were observed.

## Discussion

**Figure 2:**
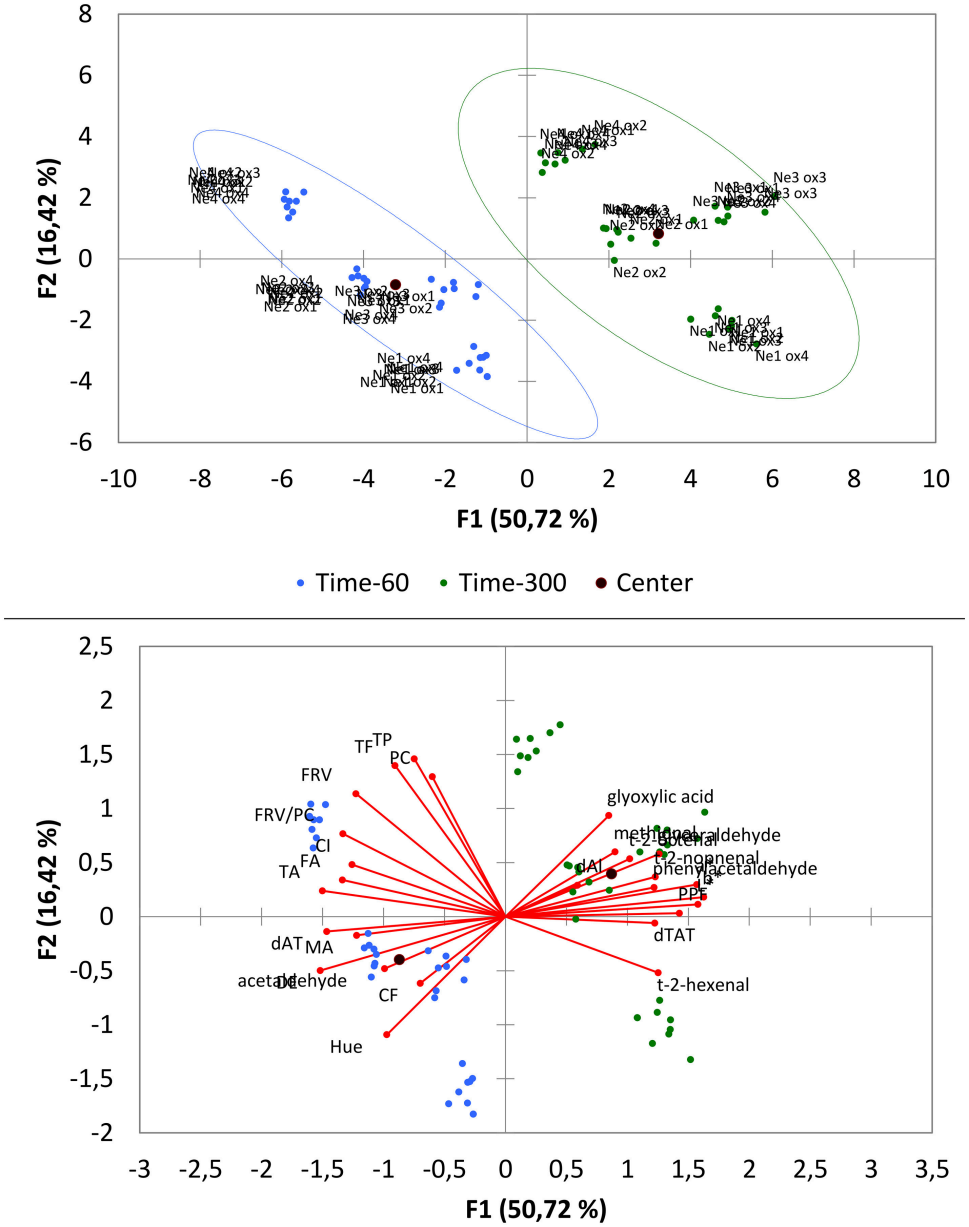
Principal Component Analysis (PCA). Representation of the wines and of the parameters related to the oxygen intake in the space defined by the first and second Principal Components after 60 (T60) and 300 (T300) days of storage using the complete dataset. Ne1-4, wines; Ox1-4, oxygen intake levels; PC, proanthocyanidins index; TP, total polyphenols index; TF, total flavonoids index; FRV, flavanols reactive to vanillin; TA, total anthocyanin index; MA, monomeric anthocyanin index; CF, copigmentation fraction; FA, free anthocyanins; PPF, polymeric pigments fraction; CI, color intensity; DE, ΔE* color parameter.

In the present study we investigated the response of different Nebbiolo wines to oxygen exposure and the subsequent impact of this initial exposure on wine aging patterns. Oxygen was applied by air stirring in sequential cycles each one involving consumption of 7 mg/L of oxygen, and analyzed after 2 months (T60). Wines were then stored in the absence of oxygen, and their composition analyzed again (T300). To gain a preliminary insight on the contribution of both initial exposure and storage on the set of analytical parameters measured at each time point, PCA was carried out on T60 and T300 all available data. The first two components explained about 67% of the total variance, with PC1 alone accounting for 50.72% of the variance (Figure 2). Separation along this PC took place based on storage time, so that top and bottom right quadrants contained wines collected at the end of the storage period (T300), which were mostly characterized by higher aldehyde concentrations (excluding acetaldehyde) and higher CIEL^*^a^*^b^*^ values, and were associated with aged wines. Left side quadrants were mostly characterized with acetaldehyde and several phenolic parameters, associated with wines at T60. Along PC2, accounting for 16.42% of the variance, samples were grouped by wine type. The changes found by the storage time are induced to the oxygen intake in the first phases of the experiment. In addition, more disperse sample groups were found at T300 with respect to T60, therefore the changes were amplified by the storage time.

The amount of oxygen supplied in the early stages of storage to young Nebbiolo wines influenced the color after 60 days of storage (Tables 1, 2). The wines with higher oxygen intake had a higher color intensity and a slightly lower hue. After 300 days of aging the color intensity decreased in all wines and the differences between the treatments within each wine remained unchanged or decreased.

The content of free and total anthocyanins decreased by increasing the oxygen supplied, the differences of concentration due to the effect of oxygen were small, even if significant in three wines out of four (except Ne4) after 60 days and in all wines after 300 days of aging. Like color intensity, the anthocyanin concentration decreased over time and the differences between wines supplied with different doses of oxygen within each wine remained unchanged.

Regarding dTAT%, a slow and gradual increase over time was observed and, after 300 days of storage, significant differences between oxygen doses were observed in two wines out of four. Coincidentally, these wines (Ne2 and Ne3) were those with the higher pH. Some authors (Morata et al., 2006) reported an increase in vitisin production in Tempranillo wines during fermentation at 20°C when the pH varied from 3.2 to 3.7. On the contrary, to our knowledge, no work has been published to date on the effect of pH on the formation of pigments during aging.

In the case of dTAT%, the differences among treatments within each wine after 60 and 300 days of aging were similar.

The only parameter that changed rapidly and significantly in all wines was the content of acetaldehyde, whose concentration after 60 days of storage significantly increased by increasing the oxygen supplied. After 300 days of storage, a significant fall in the concentration of this aldehyde was observed in all wines.

According to Di Stefano and Cravero (1989), the color of young red wines is mainly related to the monomeric anthocyanins. Over time, the contribution of pigments formed by reactions between anthocyanins and tannins, also involving molecules produced during alcoholic fermentation and/or by oxidation reactions becomes more prominent, in particular acetaldehyde. In the presence of acetaldehyde, the reactions between flavanols and anthocyanins lead to the formation of polymers made of anthocyanins and flavonols bounded with ethyl bridges (Es-Safi et al., 2002). These polymeric pigments are characterized by a higher color intensity (hyperchromic effect) and by a bathochromically shifted maximum absorption (540 nm), compared to that of monomeric anthocyanins (525 nm for malvidin-3-O-glucoside). The bathochromic shift is more accentuated in more polymerized products. These ethyl-linked pigments are protected from hydration and SO_2_ reactions (therefore not bleachable by SO_2_) by self-association phenomenon (association by sandwich-type stacking; Es-Safi et al., 2002).

Anthocyanins also participate in cycloaddition reactions with acetaldehyde and other compounds, such as pyruvic acid, vinylphenol and cumaric acid (Fulcrand et al., 1996; Schwarz et al., 2003). These reactions lead to the formation of pyranoanthocyanins and carboxypyranoanthocyanins (vitisin B and A), and phenylpyranoanthocyanins, respectively. These pigments have a red-orange color and are very stable. Like ethyl-linked pigments, they are insensitive to pH variations and not bleachable by SO_2_ (Ribéreau-Gayon et al., 2000). All these pigments are not bleachable by SO_2_ and are measured in the dTAT% fraction, whose value increased during wine aging. On the contrary, the fractions constituted by monomer anthocyanins (dAl%), and by pigments bleachable (dAT%) decreased over time (Di Stefano et al., 1989).

The presence of a high content of anthocyanins could also promote the formation of pyranoanthocyanins that are more stable molecules than the pigments between anthocyanins and tannins with ethyl bridges (Wirth et al., 2010).

Regarding to the differences in the content of acetaldehyde of the samples supplied with different doses of oxygen (T60), a larger increase of the differences of dTAT% parameter between them over time (T300) might be expected. Nevertheless, generally this did not occur in our study. These results could depend on the specific polyphenolic composition of the wines used in the trials. In fact, Nebbiolo wines are characterized by a high content of polyphenols and a low concentration of total and free anthocyanins. The condensation reactions between anthocyanins and tannins, in presence of ethyl bridges are promoted when high levels of monomer anthocyanins are available (Gómez-Plaza and Cano-López, 2011) and a 1–4 molar ratio between monomer anthocyanins and tannins (Schmidtke et al., 2011). When operating with these favorable compositional conditions, a significant increase of dTAT% parameter in Barbera wines during storage out of air after micro-oxygenation was observed (Bosso et al., 2000).

In the presence of high concentrations of polyphenol compounds, acetaldehyde could instead participate in the formation of polymers with ethylene bridges between flavanols. These polymers are characterized by UV-visible spectra similar to those of the flavan-3-ols from which they originate, with absorption maxima at 280 nm and colorless (Es-Safi et al., 1999).

Moreover, since the differences between the treatments for the color parameters appeared already during the first months of aging (T60), they probably also depend on other causes, in particular the free SO_2_ depletion caused by oxidation and/or combination with acetaldehyde.

Therefore, the production of bisulphite adducts with acetaldehyde causes the shift of the combination equilibrium between SO_2_ and monomeric anthocyanins or other pigments not bleachable by SO_2_ toward their respective colored free forms. The influence of the losses of SO_2_ on the increase of color intensity in wines exposed to different oxygen transmission rate (OTR) during bottle aging was already observed by other authors (Wirth et al., 2010; Guaita et al., 2013; Marrufo-Curtido et al., 2017). The effect of the SO_2_ losses on color intensity in Ne1 and Ne4 could result more evident due to their low pH, in accordance with the results of Kontoudakis et al. (2011) during micro-oxygenation trials.

In our work an accurate quantification of the main aldehydes present in the wine was carried out and, for the first time, the effect of increasing oxygen intakes on their evolution during the first months of storage was evaluated (Tables 5, 6).

Acetaldehyde accumulation patterns were found to be strongly wine dependent, with Ne3 and Ne4 samples showing in general higher accumulation than Ne1 and Ne2 samples. Ne4 samples were characterized by the largest increase in total acetaldehyde, in particular after consumption of the third (Ox3) and fourth (Ox4) dose of oxygen. Ne3 also attained relatively high concentrations of acetaldehyde at T60, but initial levels were already higher and therefore net accumulation was lower than Ne4. During oxidation of finished wine, acetaldehyde can be released from aldehyde-bisulfite complexes and/or be produced in the wine through oxidation of ethanol. Acetaldehyde can then react with phenolics, so that part of the released or produced acetaldehyde is then consumed by the wine itself. Differences in acetaldehyde accumulation until 60 days are therefore due to the balance between the capacity of the wine to generate acetaldehyde and its content in acetaldehyde-reactive metabolites.

Among the other aldehydes analyzed, methional also showed noticeable increases in response to the oxygen exposure cycles applied until T60. This aldehyde can be released from bisulfite complexes or arise from Strecker or Fenton reaction involving respectively methionine or methanol. Its formation is therefore connected in any case with coupled oxidation of wine ortho-diphenols, generating quinones and hydrogen peroxide involved in either SO_2_ consumption as well as Strecker and Fenton reactions (Ugliano, 2013). Nevertheless, contrary to the case of acetaldehyde and anthocyanins, we did not observe any clear relationship between the accumulation of methional and the evolution of other wine parameters.

At T300, acetaldehyde content of the wines was generally lower than at T60 (Tables S9–S12), indicating that the acetaldehyde generated upon oxidation reacted with wine components and could no longer be detected. One exception to this trend was observed for Ne2 wine, where it seemed that this acetaldehyde consuming capacity had been exhausted at T60. Only the wine obtained with the lowest degree of oxygenation was able to consume acetaldehyde between T60 and T300.

While acetaldehyde evolution emerged as a clear marker at T60 in comparison to other aldehydes, during the period T60–T300 other aldehydes were significantly impacted, in particular methional and nonenal, thought to negatively contribute to wine aroma (Culleré et al., 2007). In contrast with acetaldehyde, during T60–T300 storage, the concentration of these aldehydes generally increased markedly, although no clear relationship with previous oxygen exposure was observed. When SO_2_ is present, aldehydes other than acetaldehyde can also form reversible sulfonated adducts, which can in theory be released as free SO_2_ concentration drops (Ferreira et al., 2015). Alternatively, aldehydes such as methional or phenylacetaldehyde could be formed via Strecker reaction of oxidation-derived quinones with corresponding amino acids, or by oxidation of corresponding alcohols, with a mechanism similar to acetaldehyde formation form ethanol (Ugliano, 2013). Alkenals such as nonenal arise from the non-enzymatic oxidation of fatty acids (Escudero et al., 2002). Our data indicate that methional, nonenal, phenylacetaldehyde and to a lesser extent other alkenals, accumulate mostly during aging than during oxygen consumption, whereas the opposite is true for acetaldehyde. This could be the result of different rates of formation and decline in the wine environment, with concentration of oxidation substrates affecting the former and reactivity in bridging of phenolic compounds the latter. In any case, the observation of increases in methional and alkenals during aging is consistent with observation of higher concentrations of these compounds in commercial aged wines (Culleré et al., 2007).

## Conclusions

In this study we have described the chemical and physical changes induced by oxygen intake in four different Nebbiolo wines. The amount of oxygen supplied in the early stages of storage to young Nebbiolo wine favorably influenced the evolution of their color during 300 days of aging/storage. The wines with higher oxygen intake had a higher color intensity and a slightly lower hue.

The observed decrease in concentration of dissolved oxygen in wine, during its storage, is attributable to some important reactions involving polyphenolic compounds. These processes are essentials in the path that leads to color stabilization, and this is where the ambivalence of oxygen is best expressed. Moderate oxygenation contributes to color stabilization, while strong oxygenation of the product induces chemical reactions that could compromise wine quality.

Regarding aldehydes content, high oxygen intakes during the first phases did not markedly changed the wine concentration of these compounds at the end of the studied period, with the exception of acetaldehyde. As previously reported, this compound allows the formation of complex, colored and stable molecules, starting with the anthocyanins and the tannins present in the wine: this is the passage most concerning by the enologists. Finally, the concentration of anthocyanins and tannins and their ratio, however, can influence the amount of stable pigments that are formed in wines.

## Author contributions

Each author have substantially contributed to the realization of this research. Specifically, MP has carried out analyzes of aldehyde compounds. FT, LR co-ordinated the work, defined the experimental plan and carried out physical chemical analysis on wines. FP developed and optimized analytical methods for quantification of aldehydes, AB, MU, SG, and MP contributed substantially to the editing and review of the article and collaborate to data analysis and interpretation. All authors contributed to the manuscript and approved the final version.

### Conflict of interest statement

The authors declare that the research was conducted in the absence of any commercial or financial relationships that could be construed as a potential conflict of interest.
