# Supplementary material for: Impact of Increasing Levels of Oxygen Consumption on the Evolution of Color, Phenolic, and Volatile Compounds of Nebbiolo Wines
Source: Front Chem. 2018 Apr 27;6:137. doi: 10.3389/fchem.2018.00137 (PMC5934423; doi:10.3389/fchem.2018.00137)
Supplement: Supplementary file 1 [file Data_Sheet_1.pdf]

Table S1. General composition of wines.

	Alcohol (% v/v)	Residual sugars (g/L)	pH	Titratable Acidity (g/L)	Malic Acid (g/L)	Lactic Acid (g/L)	Tartaric Acid (g/L)	Cu (mg/L)	Fe (mg/L)
**Ne1**	14.24	3.1	3.39	5.74	0.06	1.40	1.60	< 0.10	0.86
**Ne2**	14.15	1.1	3.48	4.96	0.04	1.01	1.20	< 0.10	1.06
**Ne3**	13.60	2.0	3.55	5.27	nd	1.79	1.48	0.16	1.01
**Ne4**	13.82	1.3	3.39	5.63	nd	1.33	1.90	< 0.10	1.48

nd = not detected.

Table S2. Color characteristics of Ne1 wine during the trial.

Wine Ne1 Storage time	Oxygenation level	L*	a*	b*	Color hue	Color intensity [A.U. - O.P. 10 mm]	dTAT [%]	dAL [%]	dAT [%]	Copigmentation fraction [%]	Free anthocyanins fraction [%]	Polymeric pigments fraction [%]
T0	-	25.16 ± 0.00	54.18 ± 0.03	47.91 ± 0.00	0.88 ± 0.00	6.28 ± 0.00	28.29 ± 0.20	23.21 ± 0.23	48.50 ± 0.03	18.65 ± 0.80	30.86 ± 0.80	50.49 ± 0.00
T60	Ox1	14.97 ± 0.24d	43.67 ± 0.27c	31.87 ± 0.34c	1.16 ± 0.00d	7.56 ± 0.04a	27.11 ± 0.95a	14.97 ± 0.36	57.92 ± 0.59	12.41 ± 0.60	33.97 ± 2.67	53.62 ± 2.08
	Ox2	14.51 ± 0.05c	43.53 ± 0.03bc	31.34 ± 0.02c	1.14 ± 0.00c	7.70 ± 0.02b	28.69 ± 0.06ab	15.06 ± 0.54	56.25 ± 0.60	10.00 ± 1.58	35.59 ± 1.52	54.41 ± 0.06
	Ox3	13.92 ± 0.01b	43.09 ± 0.05ab	30.57 ± 0.04b	1.12 ± 0.00b	7.84 ± 0.01c	29.69 ± 0.77ab	13.44 ± 0.60	56.87 ± 0.17	9.61 ± 1.94	35.38 ± 1.83	55.02 ± 0.11
	Ox4	13.46 ± 0.03a	42.62 ± 0.02a	29.89 ± 0.02a	1.11 ± 0.00a	7.93 ± 0.01d	30.61 ± 1.11b	13.05 ± 0.56	56.34 ± 1.67	10.70 ± 1.46	34.51 ± 1.05	54.79 ± 0.41
T300	Ox1	25.63 ± 0.08b	54.26 ± 0.07b	49.62 ± 0.05b	0.94 ± 0.00b	6.34 ± 0.03a	40.70 ± 1.90	16.98 ± 0.15b	42.32 ± 1.74	6.37 ± 3.42	37.53 ± 7.67	56.11 ± 11.09
	Ox2	24.39 ± 0.02a	53.57 ± 0.02a	48.20 ± 0.01a	0.93 ± 0.00ab	6.61 ± 0.01b	41.97 ± 0.73	17.37 ± 0.43b	40.66 ± 0.31	12.28 ± 1.99	25.22 ± 2.66	62.50 ± 0.67
	Ox3	24.47 ± 0.06a	53.65 ± 0.05a	48.31 ± 0.10a	0.93 ± 0.00a	6.60 ± 0.01b	44.30 ± 1.64	15.19 ± 0.79a	40.51 ± 2.43	8.04 ± 2.25	30.25 ± 1.60	61.70 ± 0.65
	Ox4	24.32 ± 0.10a	53.61 ± 0.06a	48.12 ± 0.16a	0.92 ± 0.00a	6.64 ± 0.01b	43.77 ± 0.09	15.07 ± 0.35a	41.16 ± 0.26	9.88 ± 2.13	26.70 ± 3.44	63.43 ± 1.31
Sign[a] (Time)		***, ***, ***, ***	***, ***, ***, ***	***, ***, ***, ***	***, ***, ***, ***	***, ***, ***, ***	**, ***, **, ***	***, ***, ***, ***	**, ***, **, **	*, *, *, *	ns, *, ns, ns	ns, ***, ***, **
Sign[b] (Ox)		***, ***	**, ***	**, ***	***, **	***, ***	*, ns	ns, *	ns, ns	ns, ns	ns, ns	ns, ns
Sign[c] (Time × Ox)		***	***	***	***	***	ns	*	ns	ns	ns	ns

Values expressed as average ± standard deviation (n = 2). Different Latin letters within the same column and within the same time of storage indicate significant differences among oxygenation levels at $p<0.05$ (Tukey-b test). *Sign.*: *, **, *** and ns indicate significance at $p<0.05$, 0.01, 0.001 and not significant, respectively: [a] among different storage times (T0, T60, T300) within the same oxygenation level (four significances for oxygenation levels Ox1, Ox2, Ox3, and Ox4, respectively); [b] among different oxygenation levels within the same time of storage (two significances for storage times T60 and T300, respectively); [c] in a two-way ANOVA for the evaluation of the interaction of storage time and oxygenation level factors.

Table S3. Color characteristics of Ne2 wine during the trial.

Wine Ne2 Storage time	Oxygenation level	L*	a*	b*	Color hue	Color intensity [A.U. - O.P. 10 mm]	dTAT [%]	dAL [%]	dAT [%]	Copigmentation fraction [%]	Free anthocyanins fraction [%]	Polymeric pigments fraction [%]
T0	-	26.14 ± 0.04	55.02 ± 0.01	47.01 ± 0.02	0.87 ± 0.00	5.84 ± 0.01	19.17 ± 0.05	30.43 ± 0.02	50.40 ± 0.03	21.80 ± 1.56	35.91 ± 1.36	42.29 ± 0.20
T60	Ox1	16.12 ± 0.05d	44.48 ± 0.00d	32.13 ± 0.03d	1.20 ± 0.00d	7.19 ± 0.02a	22.13 ± 0.37	23.12 ± 0.50b	54.75 ± 0.13a	18.04 ± 0.68	38.72 ± 0.52	43.24 ± 0.16a
	Ox2	14.45 ± 0.03c	43.84 ± 0.01c	30.51 ± 0.05c	1.15 ± 0.00c	7.66 ± 0.03b	21.83 ± 0.68	20.21 ± 0.20a	57.96 ± 0.48b	16.47 ± 0.46	39.67 ± 0.52	43.87 ± 0.06b
	Ox3	13.48 ± 0.12b	43.07 ± 0.12b	29.35 ± 0.15b	1.12 ± 0.00b	7.91 ± 0.02c	22.22 ± 0.63	19.52 ± 0.26a	58.25 ± 0.37b	13.96 ± 0.84	41.68 ± 0.90	44.35 ± 0.06c
	Ox4	12.48 ± 0.13a	42.28 ± 0.17a	28.11 ± 0.17a	1.10 ± 0.00a	8.20 ± 0.03d	23.54 ± 0.43	17.88 ± 1.20a	58.57 ± 1.64b	13.98 ± 2.39	40.86 ± 2.32	45.16 ± 0.07d
T300	Ox1	26.37 ± 0.06c	55.39 ± 0.02b	49.62 ± 0.03c	0.91 ± 0.00c	6.11 ± 0.01a	28.26 ± 0.04a	28.76 ± 0.78b	42.98 ± 0.82	12.23 ± 1.81	30.35 ± 4.91	57.41 ± 6.72
	Ox2	24.34 ± 0.19b	54.46 ± 0.24ab	47.48 ± 0.20b	0.88 ± 0.00b	6.55 ± 0.04b	30.98 ± 0.47b	25.66 ± 0.31ab	43.36 ± 0.16	12.97 ± 3.18	33.81 ± 1.75	53.22 ± 1.43
	Ox3	23.82 ± 0.07b	54.31 ± 0.07ab	46.85 ± 0.10b	0.87 ± 0.00ab	6.67 ± 0.01b	31.81 ± 0.25b	24.59 ± 0.31a	43.60 ± 0.56	9.87 ± 0.35	36.31 ± 0.41	53.82 ± 0.06
	Ox4	22.26 ± 0.66a	53.05 ± 0.85a	44.82 ± 0.95a	0.86 ± 0.01a	7.00 ± 0.11c	34.28 ± 1.34c	22.48 ± 1.71a	43.24 ± 0.37	9.39 ± 1.23	35.53 ± 0.70	55.08 ± 0.54
Sign[a] (Time)		***, ***, ***, ***	***, ***, ***, ***	***, ***, ***, ***	***, ***, ***, ***	***, ***, ***, ***	***, ***, ***, ***	**, ***, ***, **	***, ***, ***, **	*, ns, **, *	ns, *, *, ns	ns, **, ***, ***
Sign[b] (Ox)		***, **	***, *	***, **	***, ***	***, ***	ns, **	**, *	*, ns	ns, ns	ns, ns	***, ns
Sign[c] (Time × Ox)		***	***	***	***	***	***	***	**	ns	ns	ns

Values expressed as average ± standard deviation (n = 2). Different Latin letters within the same column and within the same time of storage indicate significant differences among oxygenation levels at $p<0.05$ (Tukey-b test). *Sign.*: *, **, *** and ns indicate significance at $p<0.05$, 0.01, 0.001 and not significant, respectively: [a] among different storage times (T0, T60, T300) within the same oxygenation level (four significances for oxygenation levels Ox1, Ox2, Ox3, and Ox4, respectively); [b] among different oxygenation levels within the same time of storage (two significances for storage times T60 and T300, respectively); [c] in a two-way ANOVA for the evaluation of the interaction of storage time and oxygenation level factors.

Table S4. Color characteristics of Ne3 wine during the trial.

Wine Ne3 Storage time	Oxygenation level	L*	a*	b*	Color hue	Color intensity [A.U. - O.P. 10 mm]	dTAT [%]	dAL [%]	dAT [%]	Copigmentation fraction [%]	Free anthocyanins fraction [%]	Polymeric pigments fraction [%]
T0	-	30.05 ± 0.00	53.10 ± 0.00	47.52 ± 0.00	0.94 ± 0.00	4.95 ± 0.00	21.58 ± 0.00	25.83 ± 0.14	52.59 ± 0.14	16.79 ± 1.41	36.42 ± 1.01	46.79 ± 0.40
T60	Ox1	19.63 ± 0.22d	44.70 ± 0.04b	34.85 ± 0.13d	1.15 ± 0.01d	6.09 ± 0.04a	30.41 ± 5.52	17.84 ± 2.75b	51.75 ± 2.78	13.19 ± 2.16	38.66 ± 1.95	48.15 ± 0.21a
	Ox2	18.66 ± 0.02c	44.59 ± 0.03b	34.09 ± 0.00c	1.12 ± 0.00c	6.25 ± 0.00b	31.59 ± 9.53	15.84 ± 0.98b	52.57 ± 10.51	10.14 ± 3.19	41.14 ± 3.13	48.72 ± 0.06a
	Ox3	15.56 ± 0.29b	42.60 ± 0.31a	31.40 ± 0.33b	1.08 ± 0.00b	6.83 ± 0.05c	32.07 ± 0.88	13.39 ± 1.02ab	54.54 ± 1.90	12.10 ± 1.34	36.93 ± 1.63	50.97 ± 0.29b
	Ox4	14.90 ± 0.04a	42.19 ± 0.07a	30.65 ± 0.01a	1.07 ± 0.00a	6.95 ± 0.02d	32.16 ± 1.35	9.60 ± 0.47a	58.23 ± 1.82	12.03 ± 0.16	36.37 ± 0.04	51.60 ± 0.20b
T300	Ox1	30.99 ± 0.09d	54.89 ± 0.26c	51.93 ± 0.16d	0.95 ± 0.00c	5.06 ± 0.00a	26.56 ± 0.81a	23.31 ± 3.04b	50.14 ± 3.85	7.63 ± 0.24a	36.08 ± 3.29	56.29 ± 3.05
	Ox2	29.62 ± 0.35c	54.50 ± 0.28c	51.03 ± 0.19c	0.94 ± 0.00b	5.24 ± 0.05b	29.72 ± 0.97ab	21.14 ± 1.60b	49.15 ± 0.64	10.64 ± 1.29b	32.84 ± 2.65	56.52 ± 1.36
	Ox3	26.55 ± 0.20b	52.88 ± 0.11b	49.09 ± 0.23b	0.94 ± 0.00ab	5.75 ± 0.03c	35.80 ± 1.14bc	18.17 ± 0.25ab	46.03 ± 0.89	7.65 ± 0.28a	33.23 ± 1.03	59.12 ± 1.31
	Ox4	25.48 ± 0.07a	52.15 ± 0.07a	47.98 ± 0.14a	0.93 ± 0.00a	5.91 ± 0.00d	40.26 ± 3.30c	13.33 ± 0.20a	46.41 ± 3.50	6.62 ± 0.09a	31.57 ± 0.06	61.80 ± 0.15
Sign[a] (Time)		***, ***, ***, ***	***, ***, ***, ***	***, ***, ***, ***	***, ***, ***, ***	***, ***, ***, ***	ns, ns, ***, **	ns, **, ***, ***	ns, ns, *, *	*, ns, **, **	ns, ns, ns, **	*, **, **, ***
Sign[b] (Ox)		***, ***	***, ***	***, ***	***, **	***, ***	ns, **	*, *	ns, ns	ns, *	ns, ns	***, ns
Sign[c] (Time × Ox)		***	***	***	***	***	ns	**	ns	ns	ns	*

Values expressed as average ± standard deviation (n = 2). Different Latin letters within the same column and within the same time of storage indicate significant differences among oxygenation levels at $p<0.05$ (Tukey-b test). *Sign.*: *, **, *** and ns indicate significance at $p<0.05$, 0.01, 0.001 and not significant, respectively: [a] among different storage times (T0, T60, T300) within the same oxygenation level (four significances for oxygenation levels Ox1, Ox2, Ox3, and Ox4, respectively); [b] among different oxygenation levels within the same time of storage (two significances for storage times T60 and T300, respectively); [c] in a two-way ANOVA for the evaluation of the interaction of storage time and oxygenation level factors.

Table S5. Color characteristics of Ne4 wine during the trial.

Wine Ne4 Storage time	Oxygenation level	L*	a*	b*	Color hue	Color intensity [A.U. - O.P. 10 mm]	dTAT [%]	dAL [%]	dAT [%]	Copigmentation fraction [%]	Free anthocyanins fraction [%]	Polymeric pigments fraction [%]
T0	-	20.38 ± 0.00	52.85 ± 0.00	42.45 ± 0.01	0.74 ± 0.00	8.12 ± 0.01	20.64 ± 0.05	25.63 ± 0.47	53.72 ± 0.43	15.33 ± 2.27	45.93 ± 2.26	38.74 ± 0.01
T60	Ox1	9.61 ± 0.15d	40.22 ± 0.22d	24.42 ± 0.24c	0.98 ± 0.00a	9.71 ± 0.03a	23.91 ± 0.47	13.52 ± 1.07	62.57 ± 1.54	10.87 ± 1.74	42.23 ± 1.08	46.90 ± 0.66
	Ox2	9.26 ± 0.04c	39.67 ± 0.08c	23.98 ± 0.07c	0.99 ± 0.00c	9.95 ± 0.01b	26.10 ± 0.85	12.79 ± 1.26	61.12 ± 0.42	9.94 ± 0.23	42.84 ± 0.29	47.23 ± 0.06
	Ox3	8.81 ± 0.04b	39.07 ± 0.05b	23.24 ± 0.07b	0.99 ± 0.00bc	10.08 ± 0.01c	27.49 ± 1.36	12.42 ± 0.98	60.09 ± 2.34	8.79 ± 0.05	43.78 ± 0.08	47.43 ± 0.13
	Ox4	8.39 ± 0.02a	38.51 ± 0.03a	22.54 ± 0.04a	0.99 ± 0.00b	10.20 ± 0.01d	26.82 ± 1.80	12.21 ± 0.25	59.46 ± 0.59	8.39 ± 0.55	43.44 ± 0.69	48.17 ± 0.14
T300	Ox1	19.33 ± 0.02d	51.25 ± 0.03c	41.12 ± 0.04c	0.85 ± 0.00a	8.33 ± 0.00a	32.37 ± 0.37	18.76 ± 1.53	48.88 ± 1.90	7.65 ± 0.09	34.08 ± 3.70	58.27 ± 3.62
	Ox2	19.03 ± 0.06c	50.83 ± 0.07b	40.72 ± 0.10c	0.86 ± 0.00b	8.47 ± 0.01b	34.40 ± 0.77	17.11 ± 1.50	48.49 ± 0.73	8.86 ± 1.45	34.55 ± 0.65	56.59 ± 0.80
	Ox3	18.22 ± 0.03b	49.92 ± 0.02a	39.39 ± 0.05b	0.86 ± 0.00b	8.65 ± 0.01c	38.67 ± 4.56	16.32 ± 0.38	45.01 ± 4.18	8.92 ± 0.65	33.59 ± 0.64	57.49 ± 0.01
	Ox4	17.92 ± 0.15a	49.68 ± 0.21a	38.90 ± 0.25a	0.86 ± 0.00b	8.73 ± 0.03d	36.70 ± 1.13	15.47 ± 0.50	47.83 ± 1.63	6.82 ± 2.06	34.90 ± 2.63	58.27 ± 0.57
Sign[a] (Time)		***, ***, ***, ***	***, ***, ***, ***	***, ***, ***, ***	***, ***, ***, ***	***, ***, ***, ***	***, ***, *, **	**, **, ***, ***	**, ***, *, **	*, *, *, *	*, **, **, *	**, ***, ***, ***
Sign[b] (Ox)		***, ***	***, ***	***, ***	***, ***	***, ***	ns, ns	ns, ns	ns, ns	ns, ns	ns, ns	ns, ns
Sign[c] (Time × Ox)		***	***	***	***	***	ns	ns	ns	ns	ns	ns

Values expressed as average ± standard deviation (n = 2). Different Latin letters within the same column and within the same time of storage indicate significant differences among oxygenation levels at p<0.05 (Tukey-b test). *Sign.*: *, **, *** and ns indicate significance at p<0.05, 0.01, 0.001 and not significant, respectively: [a] among different storage times (T0, T60, T300) within the same oxygenation level (four significances for oxygenation levels Ox1, Ox2, Ox3, and Ox4, respectively); [b] among different oxygenation levels within the same time of storage (two significances for storage times T60 and T300, respectively); [c] in a two-way ANOVA for the evaluation of the interaction of storage time and oxygenation level factors.

Table S6. Phenolic content of Ne1 wine during the trial.

Wine Ne1 Storage time	Oxygenation level	Total anthocyanin index [mg malvidin-3-glucoside chloride/L]	Monomeric anthocyanins index [mg malvidin-3-glucoside chloride/L]	Total polyphenols index [mg (+)-catechin/L]	Proanthocyanidins index (PC) [mg cyanidin chloride/L]	Vanillin assay (FRV) [mg (+)-catechin/L]	FRV/PC ratio
T0	-	126.0 ± 1.1	41.2 ± 0.7	2833 ± 37	3847 ± 52	1931 ± 24	0.50 ± 0.01
T60	Ox1	116.9 ± 0.2b	23.0 ± 0.1b	2929 ± 25	3317 ± 42	1786 ± 91	0.54 ± 0.02
	Ox2	114.6 ± 0.2a	23.0 ± 0.5b	2924 ± 104	3275 ± 129	1869 ± 4	0.57 ± 0.02
	Ox3	114.0 ± 1.1a	20.2 ± 1.1ab	2968 ± 31	3349 ± 144	1838 ± 9	0.55 ± 0.03
	Ox4	112.7 ± 0.2a	19.4 ± 0.9a	2903 ± 49	3228 ± 71	1920 ± 13	0.59 ± 0.01
T300	Ox1	90.7 ± 0.2c	15.2 ± 0.2b	2929 ± 37	3414 ± 69	1557 ± 11	0.46 ± 0.01
	Ox2	89.1 ± 0.2bc	13.0 ± 0.6a	2890 ± 18	3473 ± 14	1519 ± 16	0.44 ± 0.01
	Ox3	87.0 ± 0.5a	12.8 ± 0.2a	2955 ± 25	3470 ± 73	1495 ± 18	0.43 ± 0.01
	Ox4	88.6 ± 0.9ab	13.0 ± 0.3a	2972 ± 12	3300 ± 353	1524 ± 71	0.46 ± 0.03
Sign[a] (Time)		***, ***, ***, ***	***, ***, ***, ***	ns, ns, *, ns	**, *, *, ns	*, ***, ***, **	*, **, *, ***
Sign[b] (Ox)		**, *	*, **	ns, ns	ns, ns	ns, ns	ns, ns
Sign[c] (Time × Ox)		*	**	ns	ns	ns	ns

Values expressed as average ± standard deviation (n = 2). Different Latin letters within the same column and within the same time of storage indicate significant differences among oxygenation levels at $p<0.05$ (Tukey-b test). *Sign.*: *, **, *** and ns indicate significance at $p<0.05$, 0.01, 0.001 and not significant, respectively: [a] among different storage times (T0, T60, T300) within the same oxygenation level (four significances for oxygenation levels Ox1, Ox2, Ox3, and Ox4, respectively); [b] among different oxygenation levels within the same time of storage (two significances for storage times T60 and T300, respectively); [c] in a two-way ANOVA for the evaluation of the interaction of storage time and oxygenation level factors.

Table S7. Phenolic content of Ne2 wine during the trial.

Wine Ne2 Storage time	Oxygenation level	Total anthocyanin index [mg malvidin-3-glucoside chloride/L]	Monomeric anthocyanins index [mg malvidin-3-glucoside chloride/L]	Total polyphenols index [mg (+)-catechin/L]	Proanthocyanidins index (PC) [mg cyanidin chloride/L]	Vanillin assay (FRV) [mg (+)-catechin/L]	FRV/PC ratio
T0	-	165.7 ± 0.7	73.1 ± 0.2	3215 ± 123	4128 ± 157	2578 ± 238	0.63 ± 0.08
T60	Ox1	159.9 ± 0.2c	51.6 ± 0.7c	3592 ± 55	4405 ± 195	2586 ± 67	0.59 ± 0.04
	Ox2	150.1 ± 0.0b	44.1 ± 0.7b	3566 ± 43	4338 ± 26	2597 ± 24	0.60 ± 0.01
	Ox3	148.3 ± 0.7b	41.9 ± 0.7b	3548 ± 6	4235 ± 22	2627 ± 36	0.62 ± 0.01
	Ox4	145.0 ± 1.6a	37.2 ± 2.3a	3579 ± 37	4072 ± 73	2694 ± 56	0.66 ± 0.03
T300	Ox1	114.3 ± 0.7c	25.2 ± 0.0b	3713 ± 55	4074 ± 25	2202 ± 4	0.54 ± 0.01
	Ox2	110.3 ± 0.9b	19.6 ± 3.0ab	3639 ± 25	4083 ± 2	2042 ± 36	0.50 ± 0.01
	Ox3	108.5 ± 0.2b	17.9 ± 1.6a	3683 ± 25	4134 ± 10	2221 ± 182	0.54 ± 0.04
	Ox4	106.1 ± 0.0a	16.9 ± 1.0a	3722 ± 43	4176 ± 72	2279 ± 60	0.55 ± 0.01
Sign[a] (Time)		***, ***, ***, ***	***, ***, ***, ***	*, *, **, *	ns, ns, ns, ns	ns, *, ns, ns	ns, ns, ns, ns
Sign[b] (Ox)		***, ***	**, *	ns, ns	ns, ns	ns, ns	ns, ns
Sign[c] (Time × Ox)		***	***	ns	ns	ns	ns

Values expressed as average ± standard deviation (n = 2). Different Latin letters within the same column and within the same time of storage indicate significant differences among oxygenation levels at $p<0.05$ (Tukey-b test). *Sign.*: *, **, *** and ns indicate significance at $p<0.05$, 0.01, 0.001 and not significant, respectively: [a] among different storage times (T0, T60, T300) within the same oxygenation level (four significances for oxygenation levels Ox1, Ox2, Ox3, and Ox4, respectively); [b] among different oxygenation levels within the same time of storage (two significances for storage times T60 and T300, respectively); [c] in a two-way ANOVA for the evaluation of the interaction of storage time and oxygenation level factors.

Table S8. Phenolic content of Ne3 wine during the trial.

Wine Ne3 Storage time	Oxygenation level	Total anthocyanin index [mg malvidin-3-glucoside chloride/L]	Monomeric anthocyanins index [mg malvidin-3-glucoside chloride/L]	Total polyphenols index [mg (+)-catechin/L]	Proanthocyanidins index (PC) [mg cyanidin chloride/L]	Vanillin assay (FRV) [mg (+)-catechin/L]	FRV/PC ratio
T0	-	118.2 ± 3.4	42.5 ± 0.2	3119 ± 25	3595 ± 56	2399 ± 64	0.67 ± 0.01
T60	Ox1	109.0 ± 2.3b	25.9 ± 3.7b	3475 ± 86ab	3829 ± 50b	2490 ± 38	0.65 ± 0.01a
	Ox2	107.4 ± 1.4b	22.8 ± 1.6b	3522 ± 43ab	3678 ± 59ab	2454 ± 62	0.67 ± 0.01b
	Ox3	102.7 ± 0.7ab	18.3 ± 1.1ab	3683 ± 61b	3673 ± 32ab	2386 ± 42	0.65 ± 0.01a
	Ox4	96.4 ± 3.2a	12.9 ± 0.5a	3388 ± 12a	3562 ± 7a	2408 ± 7	0.68 ± 0.01b
T300	Ox1	88.3 ± 0.5d	16.0 ± 0.1c	3488 ± 31	3764 ± 9	2210 ± 47c	0.59 ± 0.01
	Ox2	87.2 ± 0.2c	14.0 ± 0.1b	3505 ± 55	3742 ± 61	2154 ± 11bc	0.58 ± 0.01
	Ox3	85.1 ± 0.1b	9.5 ± 0.1a	3410 ± 67	3722 ± 62	2052 ± 13a	0.55 ± 0.01
	Ox4	83.6 ± 0.2a	10.0 ± 0.7a	3427 ± 6	3705 ± 11	2089 ± 13ab	0.56 ± 0.01
Sign[a] (Time)		**, **, **, **	**, ***, ***, ***	*, **, **, ***	*, ns, ns, *	*, *, **, **	**, **, **, ***
Sign[b] (Ox)		*, ***	*, ***	*, ns	*, ns	ns, *	**, ns
Sign[c] (Time × Ox)		*	***	**	ns	ns	*

Values expressed as average ± standard deviation (n = 2). Different Latin letters within the same column and within the same time of storage indicate significant differences among oxygenation levels at p<0.05 (Tukey-b test). *Sign.*: *, **, *** and ns indicate significance at p<0.05, 0.01, 0.001 and not significant, respectively: [a] among different storage times (T0, T60, T300) within the same oxygenation level (four significances for oxygenation levels Ox1, Ox2, Ox3, and Ox4, respectively); [b] among different oxygenation levels within the same time of storage (two significances for storage times T60 and T300, respectively); [c] in a two-way ANOVA for the evaluation of the interaction of storage time and oxygenation level factors.

Table S9. Phenolic content of Ne4 wine during the trial.

Wine Ne4 Storage time	Oxygenation level	Total anthocyanin index [mg malvidin-3-glucoside chloride/L]	Monomeric anthocyanins index [mg malvidin-3-glucoside chloride/L]	Total polyphenols index [mg (+)-catechin/L]	Proanthocyanidins index (PC) [mg cyanidin chloride/L]	Vanillin assay (FRV) [mg (+)-catechin/L]	FRV/PC ratio
T0	-	179.2 ± 1.4	65.7 ± 1.4	3635 ± 6	4171 ± 121	2994 ± 93	0.72 ± 0.04
T60	Ox1	154.1 ± 0.2b	30.9 ± 2.5	4211 ± 159	4175 ± 94	3268 ± 80	0.78 ± 0.01
	Ox2	148.3 ± 0.2ab	27.8 ± 2.7	4285 ± 190	4166 ± 13	3230 ± 84	0.77 ± 0.02
	Ox3	148.8 ± 3.7ab	26.0 ± 1.6	4237 ± 12	4191 ± 11	3177 ± 4	0.76 ± 0.01
	Ox4	143.8 ± 0.2a	25.4 ± 0.2	4120 ± 55	4065 ± 51	3073 ± 0	0.76 ± 0.01
T300	Ox1	115.9 ± 1.1b	19.8 ± 1.0b	4064 ± 12	4341 ± 66	2853 ± 4	0.66 ± 0.01
	Ox2	113.2 ± 0.5a	16.7 ± 1.1ab	3938 ± 67	4421 ± 16	2710 ± 51	0.61 ± 0.01
	Ox3	113.8 ± 0.0ab	16.6 ± 0.6ab	4203 ± 37	4366 ± 109	2762 ± 111	0.63 ± 0.04
	Ox4	113.2 ± 0.0a	14.0 ± 1.0a	4155 ± 116	4340 ± 128	2641 ± 78	0.61 ± 0.01
Sign[a] *(Time)*		***, ***, ***, ***	***, ***, ***, ***	*, *, ***, *	ns, ns, ns, ns	*, *, *, *	*, *, ns, *
Sign[b] *(Ox)*		*, *	ns, *	ns, ns	ns, ns	ns, ns	ns, ns
Sign[c] *(Time × Ox)*		**	ns	ns	ns	ns	ns

Values expressed as average ± standard deviation (n = 2). Different Latin letters within the same column and within the same time of storage indicate significant differences among oxygenation levels at $p<0.05$ (Tukey-b test). *Sign.*: *, **, *** and ns indicate significance at $p<0.05$, 0.01, 0.001 and not significant, respectively: [a] among different storage times (T0, T60, T300) within the same oxygenation level (four significances for oxygenation levels Ox1, Ox2, Ox3, and Ox4, respectively); [b] among different oxygenation levels within the same time of storage (two significances for storage times T60 and T300, respectively); [c] in a two-way ANOVA for the evaluation of the interaction of storage time and oxygenation level factors.

Table S10. Aroma oxygenation markers of Ne1 wine during the trial.

Wine Ne1 Storage time	Oxygenation level	Glyceraldehyde [mg/L]	Glyoxylic acid [mg/L]	Acetaldehyde [mg/L]	*t*-2-Hexenal [μg/L]	*t*-2-Octenal [μg/L]	Methional [μg/L]	*t*-2-Nonenal [μg/L]	Phenylacetaldehyde [μg/L]
T0	-	17.50 ± 0.28	0.65 ± 0.07	6.86 ± 0.22	1.55 ± 0.51	0.35 ± 0.11	3.49 ± 0.71	4.86 ± 1.07	23.39 ± 5.01
T60	Ox1	18.00 ± 0.14b	1.10 ± 0.00a	5.45 ± 0.35a	1.29 ± 0.30	0.26 ± 0.04	3.02 ± 0.36	2.76 ± 0.54	21.30 ± 3.89a
	Ox2	16.65 ± 0.07a	1.15 ± 0.07ab	6.55 ± 0.49b	1.46 ± 0.15	0.29 ± 0.08	3.29 ± 0.36	3.15 ± 0.35	26.99 ± 4.34ab
	Ox3	19.20 ± 0.14c	1.35 ± 0.07b	8.20 ± 0.14c	1.43 ± 0.35	0.32 ± 0.00	3.90 ± 0.12	3.63 ± 0.10	39.08 ± 0.84b
	Ox4	19.35 ± 0.21c	1.95 ± 0.07c	9.05 ± 0.07c	1.40 ± 0.54	0.28 ± 0.01	3.31 ± 0.05	3.38 ± 0.59	32.03 ± 4.82ab
T300	Ox1	21.70 ± 0.03a	1.49 ± 0.16a	2.55 ± 0.21a	3.34 ± 1.22	0.43 ± 0.12	4.75 ± 0.31	5.94 ± 0.11b	149.64 ± 159.91
	Ox2	20.96 ± 0.74a	1.74 ± 0.14a	3.66 ± 0.34b	1.95 ± 0.28	0.35 ± 0.01	4.26 ± 0.66	4.68 ± 0.86ab	164.27 ± 100.50
	Ox3	23.54 ± 0.37b	1.88 ± 0.06ab	2.15 ± 0.06a	2.68 ± 0.54	0.29 ± 0.02	3.21 ± 0.23	3.29 ± 0.12a	101.89 ± 2.79
	Ox4	24.00 ± 0.51b	2.20 ± 0.08b	2.32 ± 0.33a	3.70 ± 1.82	0.35 ± 0.10	3.29 ± 0.38	3.21 ± 0.05a	76.47 ± 20.92
Sign[a] *(Time)*		***, **, ***, ***	**, **, ***, ***	**, **, ***, ***	ns, ns, ns, ns	ns, ns, ns, ns	ns, ns, ns, ns	ns, ns, ns, ns	ns, ns, ***, *
Sign[b] *(Ox)*		***, **	***, *	**, *	ns, ns	ns, ns	ns, ns	ns, **	*, ns
Sign[c] *(Time × Ox)*		***	***	***	ns	ns	ns	ns	ns

Values expressed as average ± standard deviation (n = 2). Different Latin letters within the same column and within the same time of storage indicate significant differences among oxygenation levels at $p<0.05$ (Tukey-b test). *Sign.*: *, **, *** and ns indicate significance at $p<0.05$, 0.01, 0.001 and not significant, respectively: [a] among different storage times (T0, T60, T300) within the same oxygenation level (four significances for oxygenation levels Ox1, Ox2, Ox3, and Ox4, respectively); [b] among different oxygenation levels within the same time of storage (two significances for storage times T60 and T300, respectively); [c] in a two-way ANOVA for the evaluation of the interaction of storage time and oxygenation level factors.

Table S11. Aroma oxygenation markers of Ne2 wine during the trial.

Wine Ne2 Storage time	Oxygenation level	Glyceraldehyde [mg/L]	Glyoxylic acid [mg/L]	Acetaldehyde [mg/L]	*t*-2-Hexenal [μg/L]	*t*-2-Octenal [μg/L]	Methional [μg/L]	*t*-2-Nonenal [μg/L]	Phenylacetaldehyde [μg/L]
T0	-	14.60 ± 0.42	1.00 ± 0.00	3.64 ± 0.17	1.33 ± 0.27	0.45 ± 0.21	2.42 ± 0.14	4.21 ± 0.91	37.41 ± 9.48
T60	Ox1	15.25 ± 0.07	1.10 ± 0.00a	4.35 ± 0.21a	0.92 ± 0.08	0.30 ± 0.04	2.64 ± 0.09	3.31 ± 0.11	51.17 ± 2.64
	Ox2	15.70 ± 0.14	1.20 ± 0.00ab	5.30 ± 0.14b	1.05 ± 0.12	0.38 ± 0.15	3.04 ± 0.23	3.52 ± 0.37	54.68 ± 1.38
	Ox3	15.45 ± 0.49	1.40 ± 0.14b	6.30 ± 0.14c	0.81 ± 0.32	0.33 ± 0.01	2.93 ± 0.48	3.24 ± 0.75	43.87 ± 13.11
	Ox4	15.75 ± 0.49	1.80 ± 0.00c	6.45 ± 0.07c	1.10 ± 0.11	0.24 ± 0.00	2.93 ± 0.23	3.31 ± 0.11	50.90 ± 1.12
T300	Ox1	19.32 ± 0.74b	1.49 ± 0.04	2.67 ± 0.24a	2.34 ± 0.03	0.36 ± 0.00	4.99 ± 0.04	11.34 ± 0.38	72.64 ± 39.05
	Ox2	18.22 ± 0.14ab	1.61 ± 0.18	5.33 ± 0.44b	1.63 ± 0.16	0.34 ± 0.07	4.35 ± 0.18	7.78 ± 1.10	61.25 ± 29.89
	Ox3	16.64 ± 0.57a	1.81 ± 0.18	5.63 ± 0.06bc	1.84 ± 0.41	0.41 ± 0.07	4.65 ± 0.47	9.58 ± 1.54	86.21 ± 10.66
	Ox4	16.60 ± 0.11a	2.00 ± 0.03	6.31 ± 0.08c	1.55 ± 0.28	0.29 ± 0.06	4.26 ± 0.15	8.01 ± 0.05	112.85 ± 31.57
Sign[a] (Time)		**, **, ns, *	***, *, *, ***	**, *, ***, ***	**, ns, ns, ns	ns, ns, ns, ns	***, **, *, **	**, *, *, **	ns, ns, *, ns
Sign[b] (Ox)		ns, *	**, ns	***, ***	ns, ns	ns, ns	ns, ns	ns, ns	ns, ns
Sign[c] (Time × Ox)		**	**	***	ns	ns	ns	ns	ns

Values expressed as average ± standard deviation (n = 2). Different Latin letters within the same column and within the same time of storage indicate significant differences among oxygenation levels at $p<0.05$ (Tukey-b test). *Sign.*: *, **, *** and ns indicate significance at $p<0.05$, 0.01, 0.001 and not significant, respectively: [a] among different storage times (T0, T60, T300) within the same oxygenation level (four significances for oxygenation levels Ox1, Ox2, Ox3, and Ox4, respectively); [b] among different oxygenation levels within the same time of storage (two significances for storage times T60 and T300, respectively); [c] in a two-way ANOVA for the evaluation of the interaction of storage time and oxygenation level factors.

Table S12. Aroma oxygenation markers of Ne3 wine during the trial.

Wine Ne3 Storage time	Oxygenation level	Glyceraldehyde [mg/L]	Glyoxylic acid [mg/L]	Acetaldehyde [mg/L]	*t*-2-Hexenal [µg/L]	*t*-2-Octenal [µg/L]	Methional [µg/L]	*t*-2-Nonenal [µg/L]	Phenylacetaldehyde [µg/L]
T0	-	21.45 ± 0.64	0.83 ± 0.04	2.25 ± 0.01	1.28 ± 0.22	0.26 ± 0.02	10.17 ± 0.08	5.83 ± 0.14	129.03 ± 25.65
T60	Ox1	20.55 ± 0.49b	1.25 ± 0.07a	2.90 ± 0.14a	0.61 ± 0.06	0.26 ± 0.00	6.42 ± 0.08	2.25 ± 0.27	43.10 ± 7.18
	Ox2	19.25 ± 0.07b	1.30 ± 0.14a	5.50 ± 0.57b	0.47 ± 0.09	0.23 ± 0.04	6.11 ± 0.24	1.86 ± 0.10	31.26 ± 6.49
	Ox3	17.60 ± 0.42a	1.60 ± 0.00b	7.05 ± 0.07c	0.50 ± 0.01	0.33 ± 0.13	6.64 ± 0.57	1.66 ± 0.23	28.14 ± 5.09
	Ox4	19.95 ± 0.21b	1.65 ± 0.07b	12.15 ± 0.21d	0.62 ± 0.30	0.28 ± 0.05	7.32 ± 0.64	1.52 ± 0.10	34.53 ± 3.98
T300	Ox1	34.42 ± 0.76	1.86 ± 0.17	1.51 ± 0.08a	1.94 ± 0.33b	0.64 ± 0.20	14.53 ± 1.32	12.51 ± 0.84ab	216.57 ± 99.62
	Ox2	33.64 ± 0.51	1.90 ± 0.00	2.34 ± 0.35b	1.30 ± 0.11a	0.62 ± 0.12	14.77 ± 0.45	9.93 ± 1.80a	151.67 ± 77.75
	Ox3	32.84 ± 0.28	2.01 ± 0.16	2.70 ± 0.19b	2.38 ± 0.02b	1.06 ± 0.37	15.83 ± 1.73	14.74 ± 0.55b	271.59 ± 33.58
	Ox4	32.98 ± 0.25	2.23 ± 0.04	3.12 ± 0.04b	1.25 ± 0.06a	0.69 ± 0.03	12.58 ± 2.38	7.94 ± 1.37a	213.43 ± 46.81
Sign[a] (Time)		***, ***, ***, ***	**, **, **, ***	**, **, ***, ***	*, *, **, ns	ns, *, ns, **	**, ***, **, ns	***, *, ***, **	ns, ns, **, *
Sign[b] (Ox)		**, ns	*, ns	***, **	ns, **	ns, ns	ns, ns	ns, *	ns, ns
Sign[c] (Time × Ox)		*	*	***	**	ns	ns	***	ns

Values expressed as average ± standard deviation (n = 2). Different Latin letters within the same column and within the same time of storage indicate significant differences among oxygenation levels at $p<0.05$ (Tukey-b test). *Sign.*: *, **, *** and ns indicate significance at $p<0.05$, 0.01, 0.001 and not significant, respectively: [a] among different storage times (T0, T60, T300) within the same oxygenation level (four significances for oxygenation levels Ox1, Ox2, Ox3, and Ox4, respectively); [b] among different oxygenation levels within the same time of storage (two significances for storage times T60 and T300, respectively); [c] in a two-way ANOVA for the evaluation of the interaction of storage time and oxygenation level factors.

Table S13. Aroma oxygenation markers of Ne4 wine during the trial.

Wine Ne4 Storage time	Oxygenation level	Glyceraldehyde [mg/L]	Glyoxylic acid [mg/L]	Acetaldehyde [mg/L]	*t*-2-Hexenal [μg/L]	*t*-2-Octenal [μg/L]	Methional [μg/L]	*t*-2-Nonenal [μg/L]	Phenylacetaldehyde [μg/L]
T0	-	17.55 ± 0.07	0.65 ± 0.04	3.69 ± 0.13	0.72 ± 0.11	0.46 ± 0.25	3.24 ± 0.62	2.94 ± 0.37	33.58 ± 6.00
T60	Ox1	15.50 ± 0.71	1.20 ± 0.00a	2.75 ± 0.35a	0.39 ± 0.15	0.19 ± 0.04	2.23 ± 0.06	0.69 ± 0.02	16.03 ± 0.79
	Ox2	15.05 ± 0.07	1.65 ± 0.07b	5.05 ± 0.07b	0.39 ± 0.01	0.15 ± 0.01	2.58 ± 0.25	0.55 ± 0.07	10.78 ± 2.32
	Ox3	15.10 ± 0.28	1.70 ± 0.00b	8.10 ± 0.14c	0.36 ± 0.01	0.35 ± 0.21	2.65 ± 0.29	0.57 ± 0.11	10.34 ± 0.82
	Ox4	14.65 ± 0.21	1.70 ± 0.00b	10.35 ± 0.21d	0.32 ± 0.03	0.23 ± 0.05	2.74 ± 0.31	0.54 ± 0.14	15.84 ± 3.95
T300	Ox1	25.48 ± 0.34	2.20 ± 0.00	2.53 ± 0.02a	0.98 ± 0.36	0.46 ± 0.01	5.24 ± 0.96	5.12 ± 2.06	75.63 ± 54.56
	Ox2	26.94 ± 0.54	2.19 ± 0.21	3.19 ± 0.23ab	1.11 ± 0.78	0.40 ± 0.12	12.87 ± 13.04	2.98 ± 0.40	62.22 ± 45.90
	Ox3	25.68 ± 1.53	2.22 ± 0.06	3.76 ± 0.06b	0.97 ± 0.06	0.42 ± 0.01	4.85 ± 0.71	3.08 ± 0.20	68.35 ± 15.06
	Ox4	25.90 ± 0.54	2.41 ± 0.21	4.03 ± 0.40b	0.78 ± 0.20	0.41 ± 0.04	5.31 ± 0.08	4.25 ± 0.06	48.35 ± 2.09
Sign[a] (Time)		***, ***, **, ***	***, **, ***, **	*, **, ***, ***	ns, ns, **, ns	ns, ns, ns, ns	*, ns, ns, *	ns, **, **, **	ns, ns, *, *
Sign[b] (Ox)		ns, ns	***, ns	***, *	ns, ns	ns, ns	ns, ns	ns, ns	ns, ns
Sign[c] (Time × Ox)		ns	*	***	ns	ns	ns	ns	ns

Values expressed as average ± standard deviation (n = 2). Different Latin letters within the same column and within the same time of storage indicate significant differences among oxygenation levels at $p<0.05$ (Tukey-b test). *Sign.*: *, **, *** and ns indicate significance at $p<0.05$, 0.01, 0.001 and not significant, respectively: [a] among different storage times (T0, T60, T300) within the same oxygenation level (four significances for oxygenation levels Ox1, Ox2, Ox3, and Ox4, respectively); [b] among different oxygenation levels within the same time of storage (two significances for storage times T60 and T300, respectively); [c] in a two-way ANOVA for the evaluation of the interaction of storage time and oxygenation level factors.
