# Supplementary material for: Impact of Increasing Levels of Oxygen Consumption on the Evolution of Color, Phenolic, and Volatile Compounds of Nebbiolo Wines
Source: Front Chem. 2018 Apr 27;6:137. doi: 10.3389/fchem.2018.00137 (PMC5934423; doi:10.3389/fchem.2018.00137)
Supplement: Supplementary file 2 [file Data_Sheet_2.pdf]

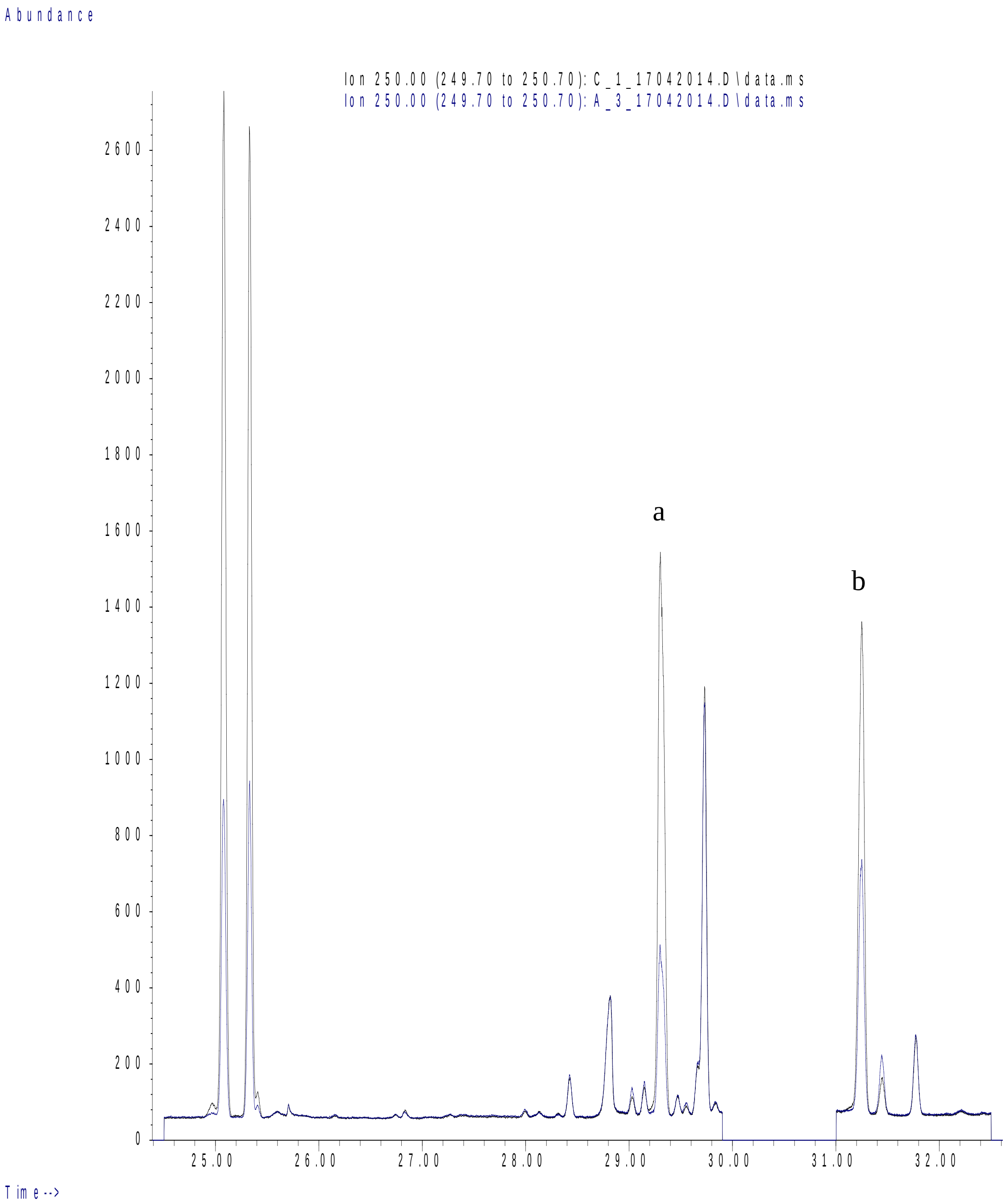

Figure S1. SIM chromatogram (target ion: 250 *m/z*) representing *t*-2-octenal (a) and *t*-2-nonenal (b) as PFBHA derivatives at two different levels of concentrations: (a) 10.4 µg/L (black line) and 2.6 µg/L (blue line); (b) 21.6 µg/L (black line) and 8.6 µg/L (blue line).

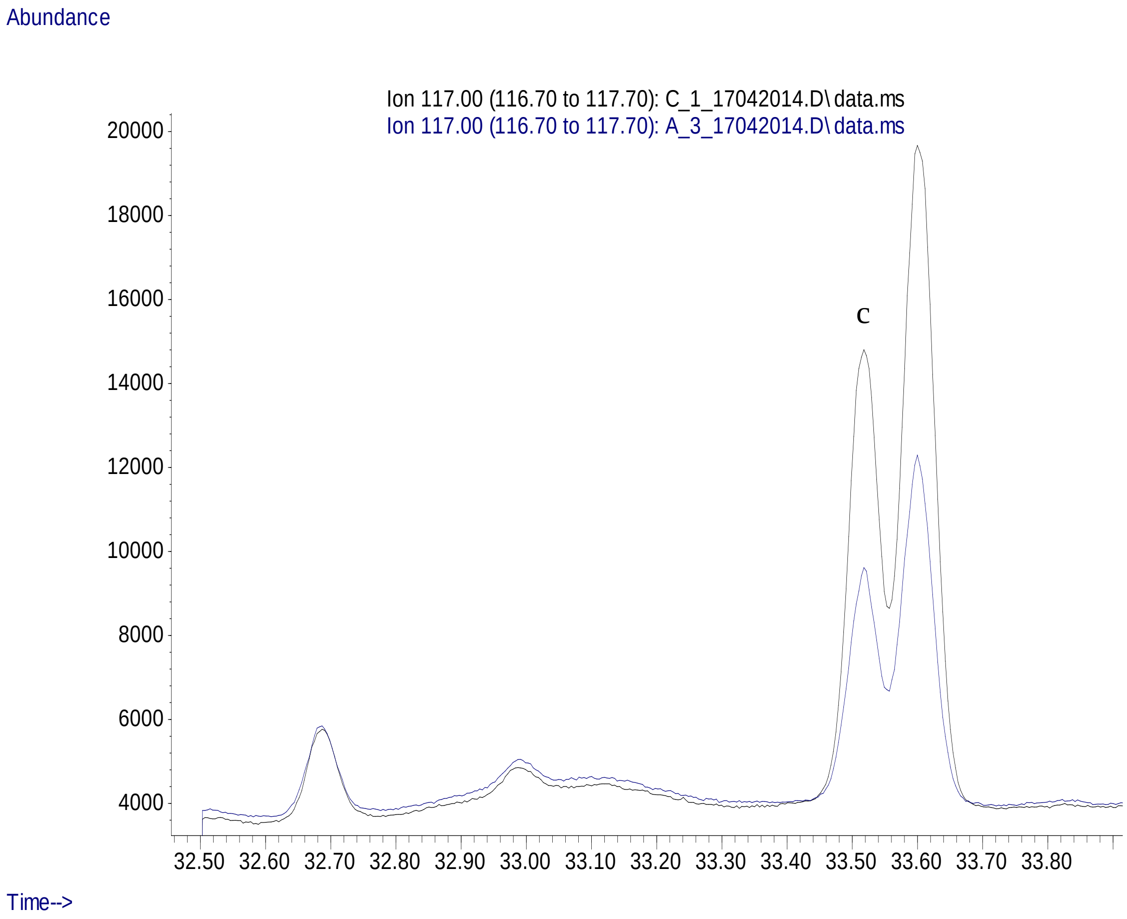

Figure S2. SIM chromatogram (target ion: 117 *m/z*) representing phenylacetaldehyde (c) as PFBHA derivative at two different levels of concentrations: 162.6 µg/L (black line) and 54.2 µg/L (blue line).

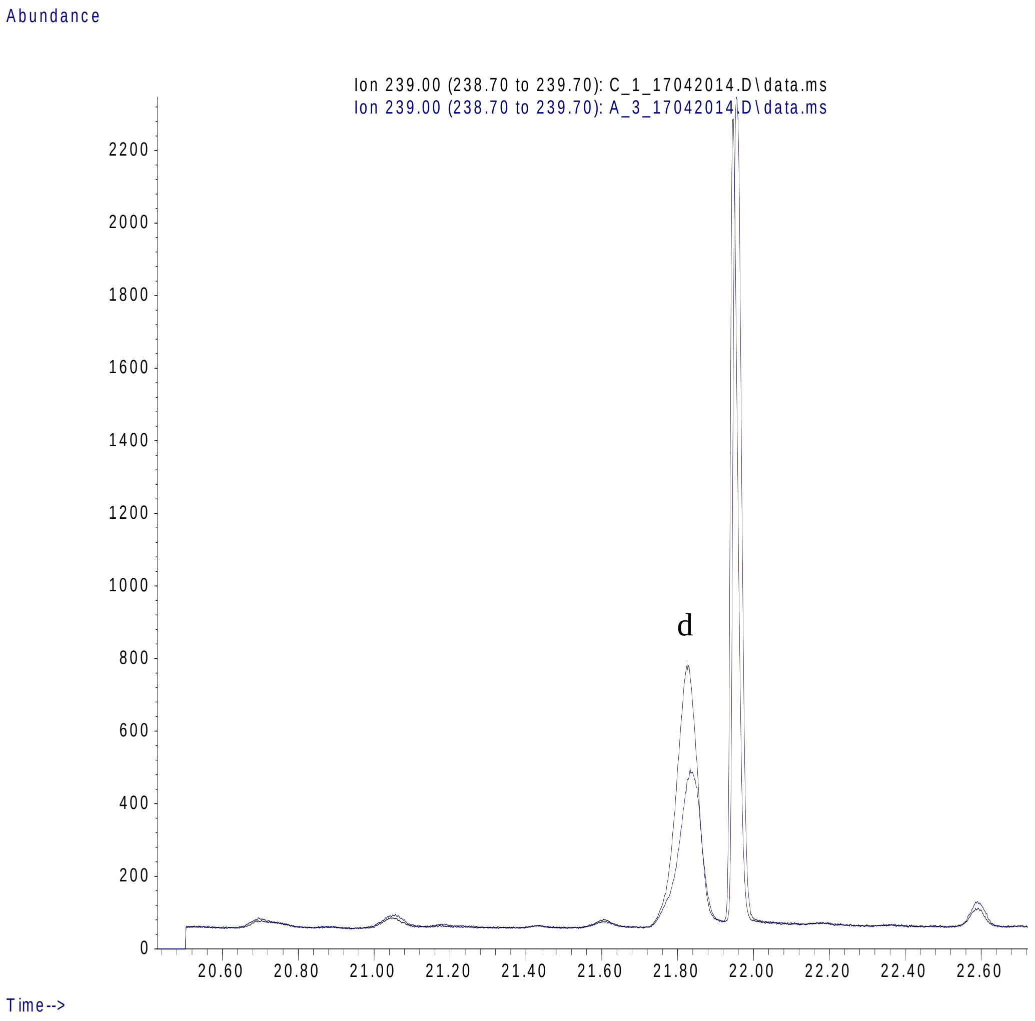

Figure S3. SIM chromatogram (target ion: 239 *m/z*) representing hexanal (d) as PFBHA derivative at two different levels of concentrations 8.45 µg/L (black line) and 2.11 µg/L (blue line).

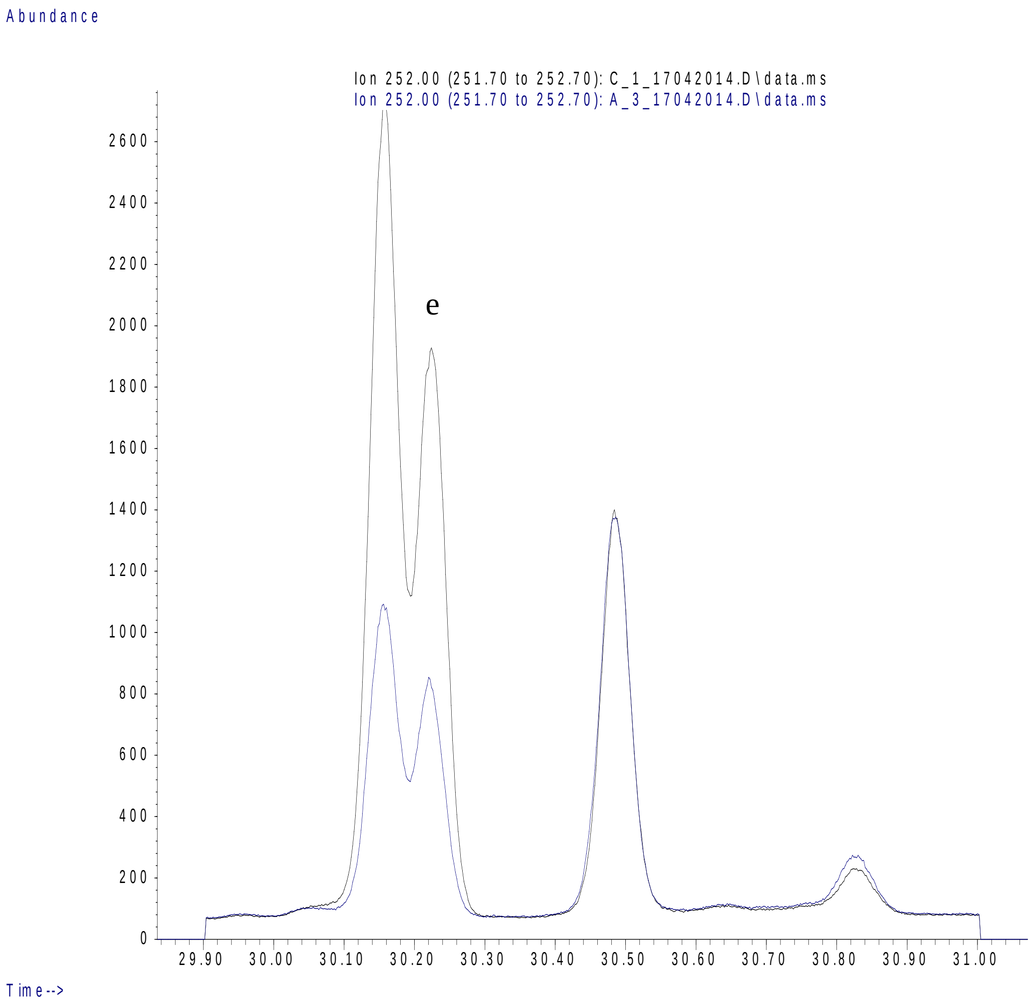

Figure S4. SIM chromatogram (target ion: 252 *m/z*) representing methional (e) as PFBHA derivative at two different levels of concentrations: 52.5 µg/L (black line) and 13.1 µg/L (blue line).

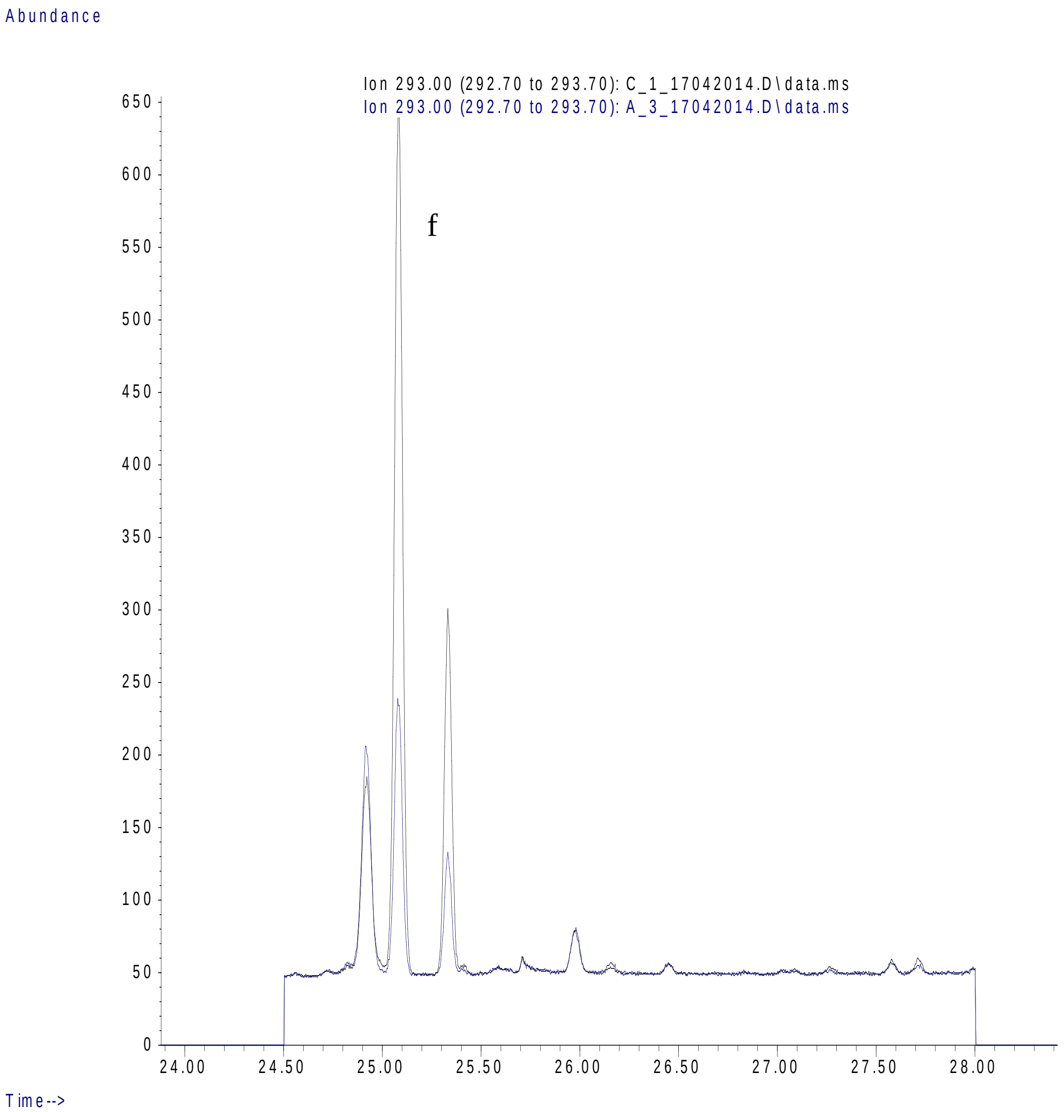

Figure S5. SIM chromatogram (target ion: 293 *m/z*) representing *t*-2-hexenal as PFBHA derivative at two different levels of concentrations: 12.5 µg/L (black line) and 3.1 µg/L (blue line).

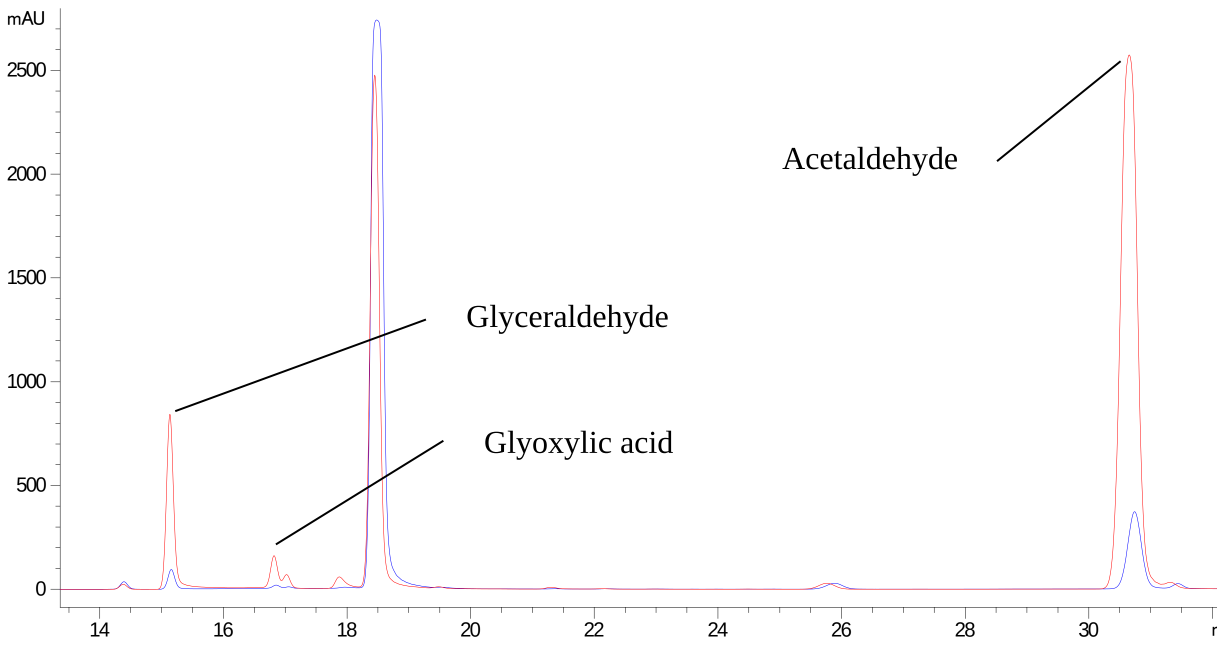

Figure S6. HPLC chromatogram representing glyceraldehyde, glyoxylic acid, acetaldehyde as DNPH derivatives at two different concentration levels.
